# Facile structuring of crystalline porous framework beads for deep purification of nuclear wastewater

**DOI:** 10.1093/nsr/nwaf080

**Published:** 2025-03-05

**Authors:** Hai-Ruo Li, Xue-Zhuo Jing, Chao-Yue Zhao, Cheng-Peng Li, Ya-Qian Lan

**Affiliations:** College of Chemistry, Tianjin Key Laboratory of Structure and Performance for Functional Molecules, Academy of Interdisciplinary Studies on Intelligent Molecules, Tianjin Normal University, Tianjin 300387, China; College of Chemistry, Tianjin Key Laboratory of Structure and Performance for Functional Molecules, Academy of Interdisciplinary Studies on Intelligent Molecules, Tianjin Normal University, Tianjin 300387, China; Ningbo Key Laboratory of Agricultural Germplasm Resources Mining and Environmental Regulation, College of Science and Technology, Ningbo University, Ningbo 315300, China; College of Chemistry, Tianjin Key Laboratory of Structure and Performance for Functional Molecules, Academy of Interdisciplinary Studies on Intelligent Molecules, Tianjin Normal University, Tianjin 300387, China; School of Chemistry, South China Normal University, Guangzhou 510006, China

**Keywords:** hydrogen-bonded organic frameworks, structuring mixed matrix beads, deep purification, ^99^TcO_4_^−^/ReO_4_^−^ sequestration

## Abstract

Traces of radionuclide residuals in ground water, with combined radiotoxicity and chemotoxicity, poses a tremendous threat to human health and the environment. Crystalline porous frameworks (CPFs), including metal-organic frameworks (MOFs), covalent organic frameworks (COFs), and hydrogen-bonded organic frameworks (HOFs), have demonstrated considerable promise as efficient adsorbents for deep purification processes. However, their microcrystalline nature often limits their practicality for industrial-scale applications. In this study, we present a facile and scalable structuring strategy to shape 17 CPFs into 34 hydrophilic and hydrophobic microbead composites using poly(acrylic acid) (PAA)-sodium alginate and polyether sulfone (PES) as co-polymers, respectively. To validate the effectiveness of this approach, the beads were employed for the sequestration of ReO_4_^−^ (a nonradioactive surrogate of ^99^TcO_4_^−^) from contaminated tap water and simulated Hanford low-activity waste (LAW). Notably, they achieved one of the highest levels of purification in treating pre-treated LAW streams, allowing purification of drinking water to nearly 5000 times their own weight under continuous flow conditions. The purified water contained only 0.026 ppb of Tc (calculated from Re), meeting both WHO (0.159 ppb) and U.S. EPA (0.053 ppb) drinking water standards. Furthermore, the beads can be conveniently and rapidly regenerated through cycling. This study provides a universal structuring strategy of CPF beads for deep purification of nuclear wastewater.

## Teaser

This study presents a facile and universal strategy for fabricating crystalline porous framework composite beads that effectively capture trace pollutants from low-level radioactive waste, offering a meaningful progress in nuclear wastewater purification.

## INTRODUCTION

**Figure 1.**
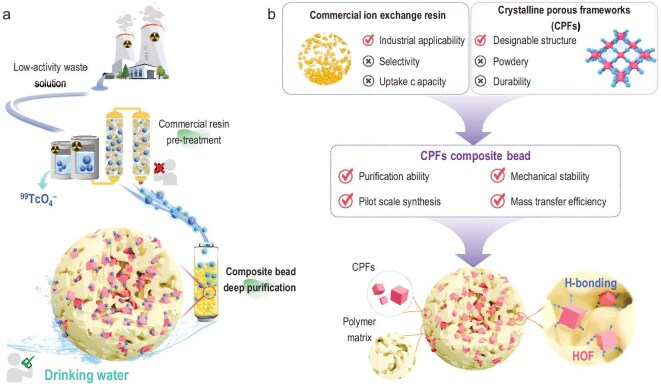
Schematic illustration of the fabrication for CPFs composite beads. (a) Deep purification of ^99^TcO_4_^−^ species in LAW streams by CPFs composite beads to produce drinking water. (b) The optimization strategy inspired by the challenges of CPFs sorbents and commercial resin in practical applications.

The importance of nuclear technology in reducing carbon emissions is growing, as the global power output of the industry is expected to rise by 55% by 2040 [1]. In a 2018 report by the Nuclear Energy Agency (NEA), a goal was set for future recycling processes in nuclear technology to achieve ‘near-zero’ radionuclide discharges, aiming to meet stringent waste management and environmental impact standards [2,3]. This objective is particularly crucial amid growing concerns over groundwater contamination at nuclear sites [4]. One significant radionuclide of concern is technetium-99 (^99^Tc), a long-lived β-emitter (half-life of 2.13 × 10^5^ years) produced in nuclear reactors during the decay of uranium-235 (^235^U). The production yield of ^99^Tc from ^235^U is ∼0.6 grams per kilogram of uranium. Annually, nearly 10 tons of ^99^Tc accumulates in the spent nuclear fuel (SNF) of global reactors. Currently, the primary technology for reprocessing SNF is the Plutonium Uranium Extraction (PUREX) process. This involves dissolving SNF in 3 M nitric acid after an initial holding period to allow short-lived radionuclides to decay. The resulting solution contains technetium in concentrations ranging from 5 to 50 ppm, corresponding to 2.2 × 10^−5^ to 2.0 × 10^−4^ mol L^−1^ of ^99^TcO_4_^−^ (the main form of ^99^Tc in solution) [5]. Due to its weak adsorption onto mineral surfaces and high solubility in water, ^99^TcO_4_^−^ is known for its significant mobility in the environment, posing challenges for wastewater management and environmental stewardship in nuclear operations [6,7]. These commercial anionic exchange resins usually possess a modest ^99^TcO_4_^−^ loading capacity in the waste streams due to excessive competitive anions and extremely aggressive environments [8]. However, these commercial resins (e.g. Purolite A532E and A530E) usually lack an effective deep purification capability towards trace ^99^TcO_4_^−^ in waste water (Fig. 1a). At the Hanford site, the most contaminated nuclear facility in the United States, ^99^Tc plumes covered 2 km^2^ of land area in 2021, with the concentrations in excess of 33.3 Bq L^−1^ (900 pCi L^−1^) of ^99^Tc [9–11].

Efficiently capturing nuclide oxyanions is crucial for developing adsorbents suitable for industrial-scale applications. While functionalized adsorbents have been continually advanced, most research has been confined to static adsorption studies, hindering their readiness for pilot-scale production. Notably, high-performance adsorbents of crystalline porous frameworks (CPFs), such as metal-organic frameworks (MOFs) [12–15], covalent organic frameworks (COFs) [16–19], porous aromatic frameworks (PAFs) [20,21], porous organic cages (POCs) [22], cationic polymer networks (CPNs) [23–25], and hydrogen-bonded organic frameworks (HOFs) [26] are typically synthesized as powders ranging from several tens of nanometers to a few microns in size. However, their powdery forms present challenges in large-scale industrial reactors. They can cause pressure drops within reactor beds, potentially leading to clogging, and are generally cumbersome to handle and recover due to their intricate separation requirements [27,28]. These factors necessitate additional costly steps in processing, limiting their practical application in industrial settings.

To address these challenges, transforming CPF powders into compacted forms with defined sizes and dimensions is essential (Fig. 1b). Shaping powders into mechanically stable objects enables them to endure high pressures and the rigorous flow conditions of gas or liquid processes [29]. Therefore, exploring the integration of CPF powdery sorbents with polymers such as polyacrylic acid (PAA), polyether sulfone (PES), polyacrylonitrile (PAN), polyetherimide (PEI), among others, holds promise [30]. By shaping these polycrystalline adsorbents, they gain enhanced mechanical strength, which streamlines separation, recovery, and transportation processes. Importantly, incorporating polymers and CPFs into pellets helps prevent excessive agglomeration of crystalline powders, mitigating risks of material leakage and secondary water contamination. This, in turn, reduces pressure drops and improves mass transfer efficiency within the adsorption column, optimizing overall performance. Therefore, after pre-treatment of commercial resin, trace ^99^TcO_4_^−^ species in the low-activity waste (LAW) streams could be deeply removed by CPF composite beads to produce drinking water expected to meet the standards set by the World Health Organization (WHO) and the U.S. Environmental Protection Agency (EPA).

**Figure 2.**
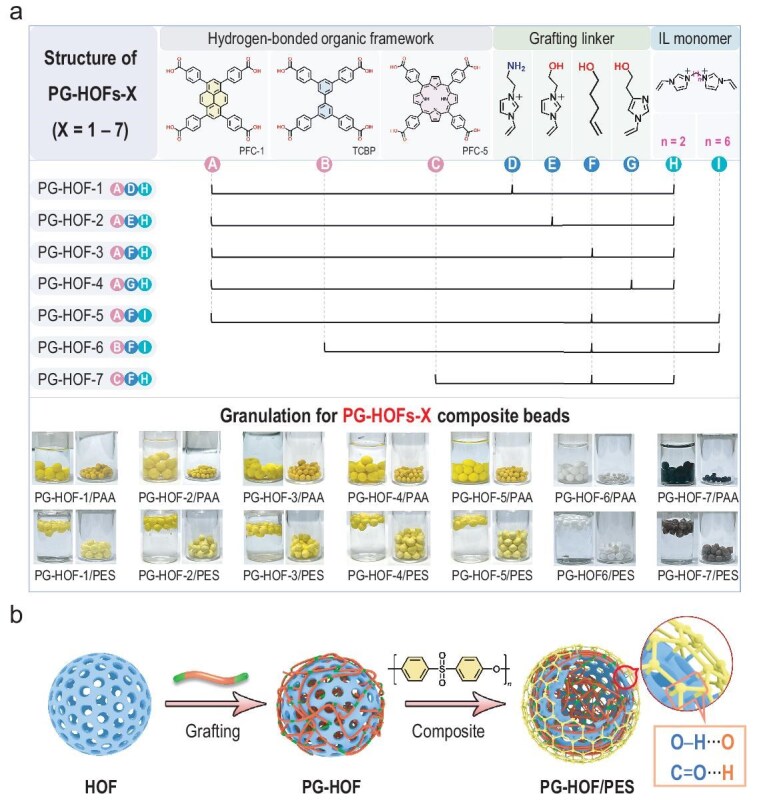
Schematic diagrams for fabrication of PG-HOFs composite beads. (a) Schematic illustration for constructing series of PG-HOFs, as well as digital photographs of 14 wet and dry PG-HOFs composite beads. (b) Schematic representation of synthesis of PG-HOF/PES composites.

In this study, we present a facile and scalable method for fabricating millimeter-sized CPF/polymer composite beads. Previously, we utilized imidazolium-based poly(ionic liquids) (PILs) to functionalize MOFs and COFs, creating P-MOFs and P-COFs for the sequestration of ^99^TcO_4_^−^/ReO_4_^−^ from nuclear wastewater [31–33]. Regarding HOFs, we introduced a novel polymerization-grafting (PG) strategy to produce ‘armor-plated’ PG-HOFs, specially designed to capture radioactive anions [26]. As a proof of concept, we successfully fabricated five P-MOFs, five P-COFs (Figs S1 and S2 in Supplementary data), and seven PG-HOFs by use of various building blocks (Fig. 2a and Figs S3–S7). These powdery CPFs were then combined with two widely used binders, polyacrylic acid (PAA) and polyether sulfone (PES), to create three distinct series of CPF/PAA and CPF/PES composite beads (Fig. 2 and Fig. S1).

To evaluate the superiority of composite beads, we selected HOFs as fillers. Unlike MOFs and COFs, HOFs consist of organic moieties connected by hydrogen bonds, which would enhance their compatibility with organic polymer matrices. Their metal-free and self-adaptive structures help reduce the interfacial gaps and promote uniform dispersion within the polymer composite beads via multiple H-bonding interactions (Fig. 2b). To this end, we chose polycrystalline PG-HOF-2 for its high uptake capacity of ReO_4_^−^ [26], despite its limitations in reusability and its application in column adsorption separations. Due to their robust mechanical stability, PG-HOF-2/PAA and PG-HOF-2/PES beads retain the advantageous properties of PG-HOF-2. They show promise as effective adsorbents for practical applications in segregating ^99^TcO_4_^−^/ReO_4_^−^ from contaminated tap water and Hanford LAW streams. These findings provide valuable insights for advancing HOFs towards real-world applications, emphasizing conditions relevant to their deployment.

## RESULTS AND DISCUSSION

### Preparation and characterization of PG-HOF-2/PAA and PG-HOF-2/PES

PG-HOF-2 powders were synthesized based on our previous work [26]. Subsequently, an aqueous solution of PG-HOF-2 and sodium alginate (SA) was added dropwise into a CaCl_2_-PAA solution, forming regular PG-HOF-2/PAA beads. PG-HOF-2/PES spherical beads were created by drop-casting a PG-HOF-2 powder/PES/*N,N*-dimethylformamide (DMF) suspension into a coagulation bath. Using similar procedures, we successfully fabricated 14 types of beads.

**Figure 3.**
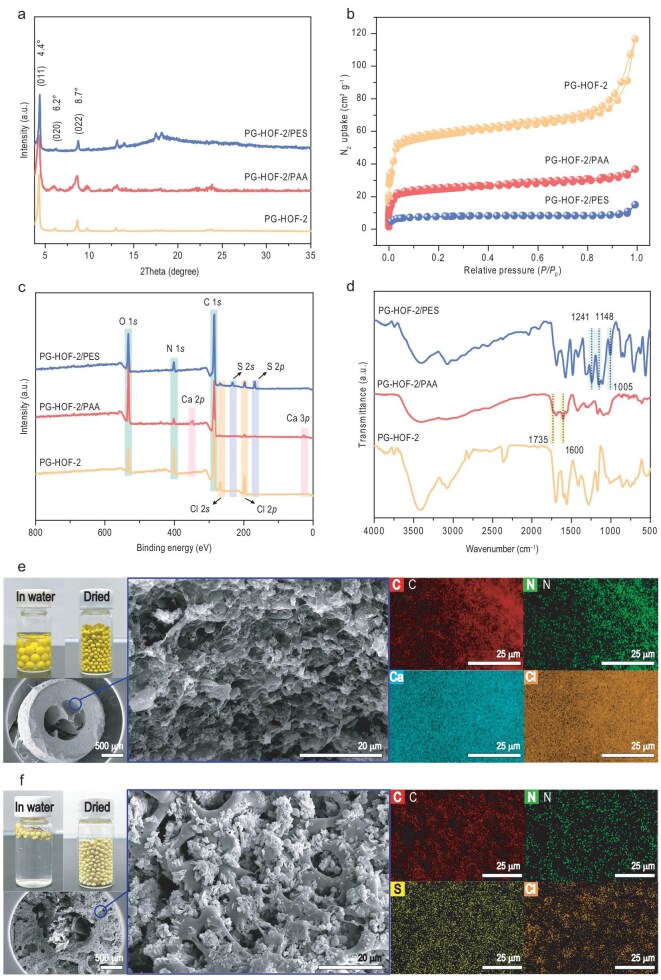
Characterization of PG-HOF-2 and its composite beads. (a) PXRD patterns. (b) N_2_ sorption isotherms. (c) XPS survey spectrum. (d) FT-IR spectra. Digital photographs and cross-sectional SEM-EDS mapping images of (e) PG-HOF-2/PAA and (f) PG-HOF-2/PES.

The powder X-ray diffraction (PXRD) patterns showed similar peaks at 4.4° (011), 6.2° (020), and 8.7° (022) for PG-HOF-2, PG-HOF-2/PAA, and PG-HOF-2/PES, indicating that the crystalline framework was retained (Fig. 3a). N_2_ adsorption isotherms at 77 K and corresponding pore size distribution revealed that the porosity of PG-HOF-2 was partially reduced in the PG-HOF-2/PAA and PG-HOF-2/PES beads due to increased mass and partially occupied pores after shaping (Fig. 3b, Fig. S8 and Table S1). Meanwhile, the ion chromatography (IC) data indicated that PG-HOF-2 contained a rich presence of Cl⁻ ions, which will facilitate ion exchange (Fig. S9). High-resolution X-ray photoelectron spectroscopy (XPS) confirmed the presence of all element signal peaks of PG-HOF-2 in both composite beads (Fig. 3c). The signal intensity of N and Cl elements in PG-HOF-2/PAA was slightly lower than in the pristine material, likely due to the introduction of calcium alginate on the PG-HOF-2 surface [34]. Additionally, Ca 2*p* and Ca 3*p* peaks appeared at 348.3 eV and 25.8 eV in PG-HOF-2/PAA, respectively. Similarly, S 2*s* (231.9 eV) and S 2*p* (168.1 eV) peaks in the XPS profiles of PG-HOF-2/PES confirmed the composite structure.

The Fourier transform infrared (FT-IR) spectra showed characteristic peaks of the imidazolium ring at 1553, 1407, and 1164 cm^−1^ in PG-HOF-2, PG-HOF-2/PAA, and PG-HOF-2/PES (Fig. 3d). In PG-HOF-2/PAA, a peak at 1604 cm^−1^ indicated coordination interactions between Ca^2+^ ions and carboxylate in PAA. In PG-HOF-2/PES, the symmetric vibration of S=O in PES was observed at 1317 cm^−1^. Thermogravimetric analysis (TGA) profiles indicated that PG-HOF-2/PES remains stable up to 300°C, comparable to PG-HOF-2, while PG-HOF-2/PAA shows poor thermal stability due to the thermolysis of cross-linking polymers. The TGA analysis revealed 61.1 wt% and 63.9 wt% of PG-HOF-2 microcrystals loaded in the PAA and PES beads, respectively (Fig. S10). Additionally, the sizes of the beads can be precisely controlled by adjusting the drip feed procedure of the PG-HOF/polymer suspension, resulting in uniform spherical beads of PG-HOF-2/PAA and PG-HOF-2/PES with a diameter of 2.00 ± 0.1 mm after heating activation for further investigation. Upon immersion in water, PG-HOF-2/PAA beads swelled due to the cross-linking molecules, while hydrophobic PG-HOF-2/PES beads floated on the liquid surface (Fig. S11).

To further investigate the distribution of PG-HOF-2 within the beads, both PG-HOF-2/PAA and PG-HOF-2/PES composites were cut in half for scanning electron microscopy (SEM) and energy-dispersive X-ray spectroscopy (EDS) characterization. SEM images showed that PG-HOF-2 particles were homogeneously embedded in the polymer matrix of PG-HOF-2/PAA beads (Fig. 3e), with Ca and Cl signals distributed throughout the beads, verifying the integration of Ca-PAA binders and PG-HOF-2 particles (Fig. S12). In PG-HOF-2/PES beads, numerous folds and finger-like cavities were observed, with PG-HOF-2 evenly distributed (Fig. 3f and Fig. S13). Importantly, the crystallinity and structure of PG-HOF-2 were preserved in all the beads mentioned. Furthermore, this structuring method can be applied to other P-MOFs, P-COFs and PG-HOFs (Figs S14–S29), demonstrating the universality of this strategy.

### Sorption performances of PG-HOF-2/PAA and PG-HOF-2/PES

**Figure 4.**
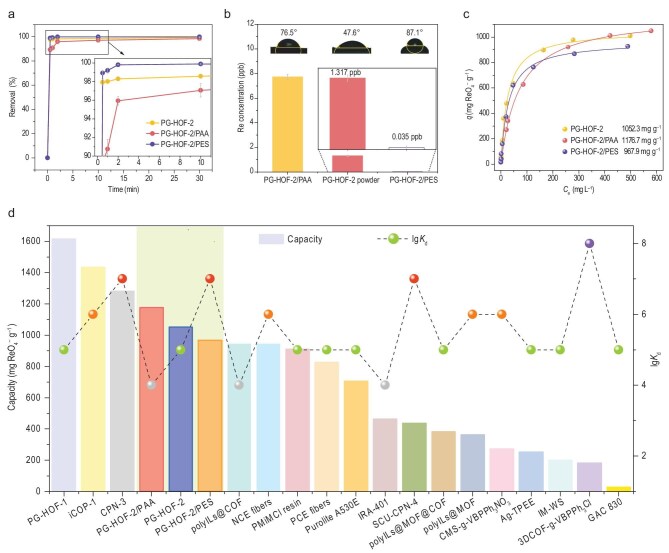
Adsorption performances of PG-HOF-2 and its composite beads. (a) Removal kinetics (initial concentration was 25 ppm Re). (b) Removal performance of various sorbents (initial concentration was 504.4 ppb Re). Inset: water contact angles of PG-HOF-2/PAA (left), PG-HOF-2 (middle), and PG-HOF-2/PES (right). (c) Langmuir isotherm curves for ReO_4_^−^. (d) Comparison of sorption capacity and *K*_d_ value (lines are guidelines for the eyes).

To evaluate the effectiveness of composite beads for ReO_4_^−^ capture, kinetic sorption experiments were carried out by immersing 10.0 mg of PG-HOF-2/PAA and PG-HOF-2/PES samples in 5.0 mL of ReO_4_^−^ solution containing 25 ppm Re at pH 7. As depicted in Fig. 4a, PG-HOF-2/PES exhibited a significantly faster sorption rate compared to PG-HOF-2/PAA and PG-HOF-2 (Figs S30, S31 and Table S2). Within the first 30 seconds, PG-HOF-2/PES removed 98.92% of ReO_4_^−^, and >99.88% was removed within 10 minutes. The kinetic data fit well with the pseudo-second-order model, demonstrating a high correlation coefficient of >0.99999 (Table S3).

Given the extremely low concentration of ^99^TcO_4_^−^ in nuclear-contaminated water, it is crucial for adsorbent materials to have excellent trapping capabilities for trace species. When treating simulated contaminated tap water containing 504.4 ppb ReO_4_^−^, PG-HOF-2, PG-HOF-2/PAA, and PG-HOF-2/PES achieved 99.74%, 98.47%, and 99.99% removal within 10 minutes, respectively (Fig. 4b). Notably, PG-HOF-2/PES reduced the residual Re concentration to 0.035 ppb. To further explain these results, we evaluated the distribution coefficient (*K*_d_) values of these materials towards ReO_4_^−^, which serve as indicators of adsorbent effectiveness [35]. The *K*_d_ values were 3.820 × 10^5^, 6.425 × 10^4^, and 1.471 × 10^7^ mL g^−1^ for PG-HOF-2, PG-HOF-2/PAA, and PG-HOF-2/PES, respectively, corresponding to the residual Re concentration of 1.317, 7.730, and 0.035 ppb (Fig. 4b). The *K*_d_(Re) value for PG-HOF-2/PES, although slightly lower than that of 3DCOF-*g*-VBPPh_3_Cl (1.0 × 10^8^ mL g^−1^) [36], is among the highest reported. Additionally, the differences in removal efficiency among the three materials are supported by their contact angles. The contact angle of PG-HOF-2/PES (87.1°) is greater than that of PG-HOF-2 (76.5°) and PG-HOF-2/PAA (47.6°), indicating the hydrophobic nature of the PES composite bead (Fig. 4b, inset). Since TcO_4_^−^/ReO_4_^−^ are less hydrophilic, the hydrophobicity of PG-HOF-2/PES enhances its selective capture of TcO_4_^−^/ReO_4_^−^ anions by overcoming the Hofmeister bias [19,24].

Encouraged by these promising results, we evaluated the sorption capacity of the PG-HOF-2 composite beads. Sorption isotherms were obtained with initial Re concentrations ranging from 50 to 800 ppm at pH 7. All sorption isotherms fit the Langmuir model well, with a high correlation coefficient of >0.99 (Figs S32, S33 and Table S4). The maximum uptake capacities for ReO_4_^−^ were 1176.7 mg g^−1^ for PG-HOF-2/PAA and 976.9 mg g^−1^ for PG-HOF-2/PES, comparable to 1052.3 mg g^−1^ for pristine PG-HOF-2 powders (Fig. 4c). In comparison, PAA and PES exhibited neglectable ReO_4_^−^ adsorption of 15.9 and 7.7 mg g^−1^ at pH 7 (Figs S34, S35). Considering both binding affinity (lg*K*_d_) and uptake capacity for ReO_4_^−^, PG-HOF demonstrated significant advantages for nuclear waste management compared to other commercial resins and reported adsorbents (Fig. 4d and Table S5).

**Figure 5.**
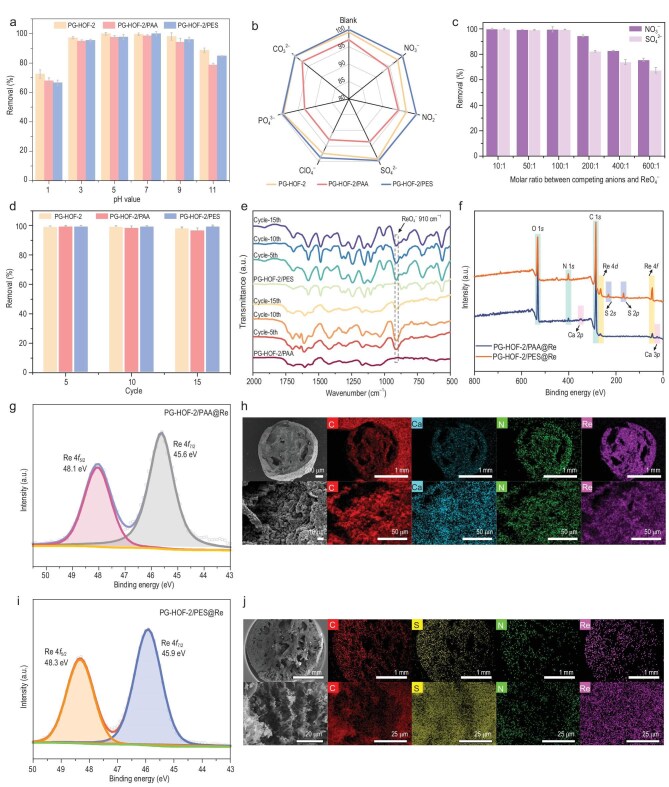
Selectivity and duration of PG-HOF-2 composite beads, and characterization of PG-HOF-2/PAA@Re and PG-HOF-2/PES@Re. (a) Effect of the pH values and (b) typical competing anions on ReO_4_^−^ removal. (c) Selectivity sorption of PG-HOF-2/PES. (d) Removal ability and (e) FT-IR spectra of PG-HOF-2/PAA and PG-HOF-2/PES during 15 cycles. (f) XPS survey of PG-HOF-2/PAA@Re and PG-HOF-2/PES@Re. Re 4*f* XPS spectra of (g) PG-HOF-2/PAA@Re and (i) PG-HOF-2/PES@Re. Cross-sectional SEM-EDS mapping images of (h) PG-HOF-2/PAA@Re and (j) PG-HOF-2/PES@Re.

The effect of pH on ReO_4_^−^ sorption by PG-HOF composite beads was tested in solutions containing 25 ppm Re at a solid-liquid ratio of 2 g L^−1^. Notably, PG-HOF-2 beads can maintain their structural integrity under pH 1–11 conditions (Fig. S36). As illustrated in Fig. 5a, PG-HOF-2 and PG-HOF-2/PES exhibited high removal percentages (>84%) for ReO_4_^−^ across a broad pH range of 3–11. By contrast, PG-HOF-2/PAA shows slightly lower removal efficiency in all cases. In radioactive contaminated water, the presence of excessive coexisting anions can significantly hinder the practical applications of TcO_4_^−^ decontamination. Therefore, we conducted sorption tests for ReO_4_^−^ by PG-HOF-2, PG-HOF-2/PAA, and PG-HOF-2/PES in the presence of equimolar concentrations of NO_3_^−^, NO_2_^−^, SO_4_^2−^, ClO_4_^−^, PO_4_^3−^, and CO_3_^2−^. As shown in Fig. 5b, PG-HOF-2/PES showed superior performances (>98% removal) compared to PG-HOF-2 and PG-HOF-2/PAA in all cases (Table S6). Given that sulfate and nitrate levels in groundwater are much higher than the concentration of ^99^TcO_4_^−^, we further tested the sorption capabilities of PG-HOF-2/PES with varying molar ratios of NO_3_^−^/SO_4_^2−^ to ReO_4_^−^, ranging from 1 to 100. All the removal efficiencies remained ∼100% (Fig. 5c). Even at a ratio of 600:1, PG-HOF-2/PES was able to sequester 75.8% and 67.4% of ReO_4_^−^, respectively. These results demonstrate that PG-HOF-2/PES has high selectivity and strong affinity for ReO_4_^−^/TcO_4_^−^, even in the presence of large excesses of NO_3_^−^ and SO_4_^2−^, likely due to its hydrophobic nature.

Reusability of sorbents is a crucial parameter, significantly reducing overall costs and enhancing the sustainability of TcO_4_^−^ disposal processes. We assessed the reusability through static ReO_4_^−^ adsorption/desorption tests. The adsorbent powders or beads were immersed in a 25 ppm Re aqueous solution and then regenerated with a 3 M HCl solution. As a result, PG-HOF-2, PG-HOF-2/PAA, and PG-HOF-2/PES demonstrated >97% ReO_4_^−^ removal and structural integrity even after 15 adsorption/desorption cycles (Fig. 5d and Fig. S37). Remarkably, the *K*_d_ value of PG-HOF-2/PES was as high as 1.16 × 10^7^ mL g^−1^ after the 15th cycle (Table S7). FT-IR spectra revealed a new peak at 910 cm^−1^ after anion exchange, corresponding to the Re–O stretching vibrations of ReO_4_^−^, confirming the formation of PG-HOF-2/PAA@Re and PG-HOF-2/PES@Re (Fig. 5e). Additionally, the detection of the Re element and the decrease of Cl in the survey spectrum of PG-HOF-2/PAA@Re and PG-HOF-2/PES@Re further confirmed ReO_4_^−^ sorption via anion exchange (Fig. 5f and Table S8). In the XPS survey spectra, the binding energies of Re 4*f*_7/2_ exhibited a red shift from 46.1 eV for KReO_4_ [34] to 45.6 eV for PG-HOF-2/PAA@Re and 45.9 eV for PG-HOF-2/PES@Re (Fig. 5g and i). This shift indicates a strong interaction between ReO_4_^−^ and imidazolium, resulting in increased electron density. Cross-sectional SEM-EDS mapping images of PG-HOF-2/PAA@Re and PG-HOF-2/PES@Re further confirmed the anion exchange of Cl^−^ by ReO_4_^−^ (Fig. 5h and j). We have analyzed the variation in the binding energy of the N 1s peak in PG-HOF-2/PAA@Re (PG-HOF-2/PAA) and PG-HOF-2/PES@Re (PG-HOF-2/PES). The binding energy of N^+^ (–C–N^+^–C–) in imidazolium shifted from 401.5 eV (in PG-HOF-2/PAA and PG-HOF-2/PES) to 401.9 eV (in PG-HOF-2/PAA@Re and PG-HOF-2/PES@Re). In addition, a new peak corresponding to N^+^–O^−^ appeared at 399.9 eV for PG-HOF-2/PAA@Re and 400.0 eV for PG-HOF-2/PAA@Re. This indicates that the N^+^ in imidazolium group interacts with ReO_4_^−^ in both PG-HOF-2/PAA and PG-HOF-2/PES (Figs S38 and S39). Notably, distribution of Ca and S elements was homogeneous in PG-HOF-2/PAA@Re and PG-HOF-2/PES@Re, while the Re distribution matched that of N in both beads, indicating that PG-HOF-2 particles were primarily responsible for capturing ReO_4_^−^ rather than the PAA or PES polymers.

### Dynamic sorption column tests on simulated contaminated tap water and LAW stream

**Figure 6.**
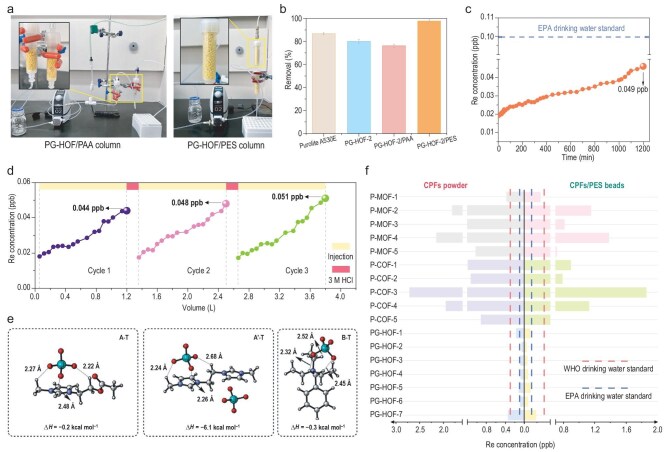
Dynamic sorption columns tests. (a) Digital photograph of PG-HOF-2/PAA and PG-HOF-2/PES fixed-bed column adsorption apparatus. (b) Comparison of Purolite A530E, PG-HOF-2, PG-HOF-2/PAA, and PG-HOF-2/PES for ReO_4_^−^ sorption in simulated LAW stream. (c) Dynamic sorption of PG-HOF-2/PES column for pre-treated LAW solution. (d) Reusability performance of PG-HOF-2/PES during three cycles (pink gaps represent regeneration process). (e) Optimized geometries of the adsorption complexes of **A-T, A′-T** and **B-T**. Numbers in black are hydrogen bond distances (Å) between atoms. The adsorption enthalpies (∆*H*) are measured with respect to the TcO_4_^−^ anion and the adsorbent material (**A, A'**, or **B**). (f) Adsorption ability of CPFs-X and CPFs-X/PES for pre-treated LAW solution. Due to the radioactivity of ^99^Tc, Re species was used throughout the work. The WHO (0.159 ppb) and U.S. EPA (0.053 ppb) standards for ^99^Tc in drinking water amount to 0.299 and 0.100 ppb of Re, highlighted with red and blue dashed lines in the diagram.

From a practical application viewpoint, structuring the sorbent is crucial for continuous flow conditions [37]. Shaping PG-HOF into beads can meet engineering requirements, indicating their potential in sustainable recovery systems. To demonstrate the effectiveness of PG-HOF composite beads for TcO_4_^−^ capture and recovery, we assembled dynamic sorption columns filled with PG- HOF-2/PAA and PG-HOF-2/PES (Fig. 6a). In the PG-HOF-2/PES column, quartz sand was added to prevent flotation and aggregation of the hydrophobic sorbent during wetting (Fig. S40). Due to the swelling nature of PG-HOF-2/PAA beads in water, a gas sparging system was implemented by injecting a gas line into the column inlet, enhancing mass transfer within the anion exchange system (Fig. S41). As shown in Figs S42 and S43, we pumped simulated contaminated tap water containing 20 ppm Re through 1.0 g PG-HOF-2/PAA beads and PG-HOF-2/PES beads (0.5 g bead per column) at a flow rate of 1.0 mL min^−1^ for dynamic sorption. Taking 95% Re removal as the breakthrough point, PG-HOF-2/PAA could effectively remediate 250.0 mL of Re solution. In comparison, the PG-HOF-2/PES column could process 900.0 mL of solution before reaching the breakthrough point.

The impressive results from our initial tests prompted us to use a simulated Hanford LAW melter recycle stream, spiked with ReO_4_^−^, as a surrogate for radioactive ^99^TcO_4_^−^ (Table S9). Under static anion exchange conditions, Purolite A530E, PG-HOF-2 powder, PG-HOF-2/PAA, and PG-HOF-2/PES removed 86.72%, 80.23%, 76.13%, and 97.74% of ReO_4_^−^ from LAW simulated solutions, respectively (Fig. 6b). To further evaluate the performance of PG-HOF-2 composite beads in removing trace amounts of ReO_4_^−^, we pre-treated the simulated LAW stream with commercial A530E resin. The resulting Purolite A530E-treated effluent, containing 12.298 ppb of Re, was then passed through adsorption columns filled with Purolite A530E resin, PG-HOF-2/PAA, and PG-HOF-2/PES, respectively. The residual Re concentrations in the effluent were 8.498 ppb for Purolite A530E and 5.479 ppb for PG-HOF-2/PAA at a flow rate of 1.0 mL min^−1^ over 12 hours (Fig. S44). In contrast, 1 g of PG-HOF-2/PES effectively purified 1200.0 mL of pre-treated LAW solution per cycle, reducing the residual Re concentration to only 0.049 ppb (Fig. 6c). This corresponds to 0.026 ppb of Tc, fully meeting both WHO (0.159 ppb) and U.S. EPA (0.053 ppb) standards for ^99^Tc in drinking water. The PG-HOF-2/PES column, regenerated using a 3 M HCl eluent, was reused for at least three consecutive cycles (Fig. 6d). Consequently, 1.0 g of PG-HOF-2/PES beads could treat 4.8 L of contaminated water from primary processes.

The exceptional purification capability of PG-HOF-2/PES can be properly elucidated by density functional theory (DFT) calculations [26]. The optimized structures and electrostatic potential distributions of the PG-HOF-2 fragments (A and A') and the Purolite A530E fragment (B) are show in Fig. S45. Notably, the maximum positive potentials in fragments A (+4.6 eV) and A' (+6.9 eV) are both located near the imidazole ring, and are higher than that in fragment B (+4.5 eV). This indicates that the imidazolium groups in PG-HOF-2 offer a stronger electrostatic attraction to the negatively charged TcO_4_^−^ (−5.1 eV) than the quaternary ammonium groups in Purolite A530E resin. Additionally, multiple C–H···O hydrogen bonds form between the sorbents and TcO_4_^−^ (Fig. 6e). The calculated adsorption enthalpy (Δ*H*) values for A-T (−7.1 kcal/mol) and A'-T (−6.1 kcal/mol) are significantly higher than for B-T (−0.3 kcal/mol), clearly demonstrating that PG-HOF-2 is more favorable to sequester trace TcO_4_^−^ than quaternary ammonium-type commercial resins. Furthermore, we analyzed the purification efficiency of each CPF-X powder and CPF-X/PES for the pre-treated LAW solution (Fig. 6f and Table S10). The CPF-X/PES beads consistently outperformed their parent CPF-X powders in deep purification. Notably, both the PG-HOFs/PES and P-MOF-1/PES beads successfully purified the LAW solution to meet the safety standards set by the WHO.

## CONCLUSION

To date, the exploration of CPF-based materials for water purification under continuous liquid flow remains limited. One major challenge is the poor stability of CPF in water, compounded by the lack of straightforward structuring methods that maintain the properties of the parent CPF material. Addressing this, we developed a facile and scalable microdroplet approach to fabricate millimeter-scale porous CPF-based composite beads by combining functionalized CPFs with PAA or PES macromolecules. The universality of this simple structuring method was demonstrated using a variety of structurally diverse P-MOFs-X (X = 1–5), P-COFs-X (X = 1–5), PG-HOFs-X (X = 1–7), resulting in the production of 34 different functionalized CPF composite beads. Due to the mild nature of this shaping method, the structure and crystallinity of all CPFs were well preserved in the beads. This approach not only ensures the stability of CPFs in water but also retains their advantageous properties for effective water purification applications.

To assess the applicability of our structuring method for remediating nuclear waste water, we conducted a proof-of-concept study using PG-HOF-2, known for its exceptional performance in trapping ^99^TcO_4_^−^/ReO_4_^−^ from water. PG-HOF-2 was structured into hydrophilic PG-HOF-2/PAA and hydrophobic PG-HOF-2/PES beads. Both types of beads demonstrated impressive ReO_4_^−^ uptake capacities of ∼1 g g^−1^. However, the hydrophobic PG-HOF-2/PES beads exhibited rapid adsorption kinetics (<30 seconds) and superior binding affinity for ReO_4_^−^ (*K*_d_ = 1.471 × 10^7^ mL g^−1^). Notably, 1 g of PG-HOF-2/PES beads effectively purified 4.8 L of pre-treated simulated LAW solution. That is, the PG-HOF-2/PES beads can purify drinking water nearly 5000 times its own weight under continuous flow conditions. As a result, the PG-HOF-2/PES beads efficiently reduced trace Re concentrations to 0.049 ppb, being equal to 6‰ of that of commercial resin (residual concentration: 8.498 ppb), well below the WHO (0.159 ppb) and U.S. EPA (0.053 ppb) thresholds for drinking water. This demonstrates the potential of PG-HOF-2/PES beads in producing potable water from nuclear wastewater.

In conclusion, this work presents a straightforward and scalable method for fabricating CPF-based composite beads aimed at sequestering ^99^TcO_4_^−^ from nuclear waste. This approach effectively addresses challenges related to the separation and recovery of powdery CPF adsorbents in practical applications. We anticipate that this method can be easily adapted for numerous other CPFs and their composites, thereby facilitating the broader utilization of CPFs across various large-scale applications.

## METHODS

Preparation of CPF/PAA beads: 800.0 mg of CPF powder samples were dispersed in 15.0 mL of sodium alginate (SA) aqueous solution. Meanwhile, 1.1504 g of CaCl_2_ were dissolved in 200.0 mL of polyacrylic acid (PAA, Mw = 2000) aqueous solution, which served as the curing solution. Subsequently, the CPF/SA suspensions were added into the curing solution using a syringe. The resultant spherical CPF/PAA beads were collected and thoroughly washed with water, and then activated upon heating to 80°C under vacuum.

Preparation of CPF/PES beads: 1.0012 g of polyether sulfone (PES) were fully dissolved in 10.0 mL of DMF through mechanical stirring for 24 hours at 60°C, yielding the transparent PES solution with uniform viscosity. Then 800.0 mg of CPF powder was added to the PES solution under stirring for overnight. The suspensions were added dropwise by syringe into a precooled water/ethanol (v/v = 1:1) solution. As a result, CPF/PES beads were immediately formed by solvent/water exchange. The resultant beads were collected and thoroughly washed with water and ethanol, and then activated upon heating to 80°C under vacuum.

## Supplementary Material

nwaf080_Supplemental_File
