# Correction to: Facile structuring of crystalline porous framework beads for deep purification of nuclear wastewater

**DOI:** 10.1093/nsr/nwaf234

**Published:** 2025-06-18

**Authors:** Hai-Ruo Li, Xue-Zhuo Jing, Chao-Yue Zhao, Cheng-Peng Li, Ya-Qian Lan

**Affiliations:** College of Chemistry, Tianjin Key Laboratory of Structure and Performance for Functional Molecules, Academy of Interdisciplinary Studies on Intelligent Molecules, Tianjin Normal University, Tianjin 300387, China; College of Chemistry, Tianjin Key Laboratory of Structure and Performance for Functional Molecules, Academy of Interdisciplinary Studies on Intelligent Molecules, Tianjin Normal University, Tianjin 300387, China; Ningbo Key Laboratory of Agricultural Germplasm Resources Mining and Environmental Regulation, College of Science and Technology, Ningbo University, Ningbo 315300, China; College of Chemistry, Tianjin Key Laboratory of Structure and Performance for Functional Molecules, Academy of Interdisciplinary Studies on Intelligent Molecules, Tianjin Normal University, Tianjin 300387, China; School of Chemistry, South China Normal University, Guangzhou 510006, China

In the Fig. 3 of ‘Facile structuring of crystalline porous framework beads for deep purification of nuclear wastewater’ (*National Science Review*, Volume 12, Issue 5, 2025, nwaf080, https://doi.org/10.1093/nsr/nwaf080), the data for FT-IR spectra of PG-HOF-2 and its composite beads were incorrectly provided (Fig. 3d). The corrected version of Fig. 3 is presented below.

**Figure 3.**
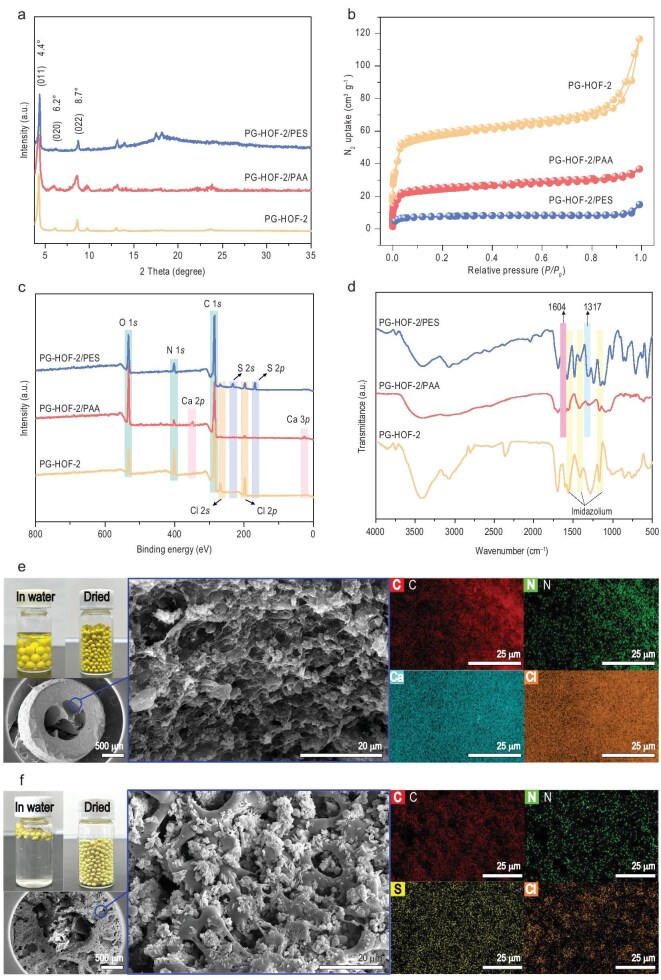
Characterization of PG-HOF-2 and its composite beads. (a) PXRD patterns. (b) N_2_ sorption isotherms. (c) XPS survey spectra. (d) FT-IR spectra. Digital photographs and cross-sectional SEM-EDS mapping images of (e) PG-HOF-2/PAA and (f) PG-HOF-2/PES.

