# Supplementary material for: Facile structuring of crystalline porous framework beads for deep purification of nuclear wastewater
Source: Natl Sci Rev. 2025 Mar 5;12(5):nwaf080. doi: 10.1093/nsr/nwaf080 (PMC11983684; doi:10.1093/nsr/nwaf080)
Supplement: nwaf080_Supplemental_File [file nwaf080_supplemental_file.pdf]

Supporting Information for

Facile Structuring of Crystalline Porous Framework Beads for Deep Purification of Nuclear Wastewater

Hai-Ruo Li,[1] Xue-Zhuo Jing,[1] Chao-Yue Zhao,[2] Cheng-Peng Li,[1,*] and Ya-Qian Lan[3,*]

[1]College of Chemistry, Tianjin Key Laboratory of Structure and Performance for Functional Molecules, Academy of Interdisciplinary Studies on Intelligent Molecules, Tianjin Normal University, Tianjin 300387, China

[2]Ningbo Key Laboratory of Agricultural Germplasm Resources Mining and Environmental Regulation, College of Science and Technology, Ningbo University, Zhejiang 315300, China

[3]School of Chemistry, South China Normal University, Guangzhou 510006, China

Section S1. Experimental Section

1. Materials and chemicals

All the chemical reagents were analytical grade and directly used without further purification unless otherwise specified. 1,3,6,8-tetrakis(benzoic acid)pyrene (H_4TBAPy), 3,3’,5,5’-tetrakis-(4-carboxyphenyl)-1,1’-biphenyl (H_4TCBP), and 5,10,15,20-tetrakis(4-carboxyphenyl) porphyrin (H_4TCPP) were purchased from Yanshen Technology Co., Ltd (Jilin, China). 2,2’-azobis(isobutyronitrile) (AIBN) were from Meryer (Shanghai) Chemical Technology Co. Ltd. 1-[3-(dimethylamino)propyl]-3-ethylcarbodiimide hydrochloride (EDC) were purchased from Shanghai Macklin Biochemical Co., Ltd. Potassium perrhenate ($KReO_4$) were got from Shanghai Tensus Biotech Co., Ltd.

2. Characterizations

Powder X-ray diffraction (PXRD) patterns were collected on a Bruker D8 Advance diffractometer with Cu-Kα radiation (λ = 1.5418 Å), operating at 40 kV and 100 mA, and the diffraction intensity data were obtained by the continuous scans in the $2\theta/\theta$ mode with the scan rate of 2 s/step and step size of 0.02°. X-ray photoelectron spectroscopy (XPS) was taken on a Thermo Scientific ESCALAB Xi+ spectrometer. FT-IR spectra were recorded on a Bruker AlPHA spectrometer in a range of 4000–500 cm^{-1}. Nitrogen sorption isotherms were recorded at 77 K on Micromeritics ASAP 2020 HD88 apparatus. Energy dispersive X-ray spectroscopy (EDS) was conducted on JEOL JEM-F200 with the acceleration voltage of 200 kV. Scanning electron microscope (SEM) was taken on TESCAN MIRA LMS. HRTEM images were taken by a JEOL JEM-2100 plus equipped with a TVIPS XF416 camera. The Re concentrations in solution were determined on a Perkin Elmer NexION 350D inductively coupled plasma mass spectrometer (ICP-MS) after degradation of the sample in HNO_3. Thermogravimetric analysis (TGA) was taken on TGA Q500 thermal analyzer in 25–800 ℃ at a heating rate of 20 ℃ min^{-1} under N_2 atmosphere.

The water contact angle (CA) was measured on Dataphysics OCA40. Ion chromatography (IC) measurement was taken on Dionex ICS-1000.

3. Syntheses

Synthesis of P-MOFs-X (X = 1–5). All of the MOFs and ionic liquids were prepared according to our previous work.[1] Activated MIL-101 (2.0000 g) and 3,3’-divinyl-1,1’(1,2-ethanediyl)diimidazolium dibromide (*bis*-C_2, 1.0000 g) in CH_3OH (40.0 mL) were stirred at room temperature for 24 hours. Then, 2,2’-azobis(isobutyronitrile) (AIBN, 250.0 mg) in 5.0 mL CH_3OH was added, which was heated to 70 ℃ under N_2 atmosphere for 24 hours. After that, additional 5.0 mL CH_3OH solution of AIBN (250.0 mg) was added, which was heated at 70 ℃ under N_2 atmosphere for 48 hours. The green solids were collected and washed with DMF and methanol for three times, and dried at 50 ℃ under vacuum. After immersing in NaCl solution for 3 days, the powdery materials were dried under vacuum overnight to produce P-MOF-1. P-MOF-2 and P-MOF-3 were synthesized by the similar procedure to that of P-MOF-1, except *bis*-C_2 was replaced by 1-vinyl-3-ethylimidazolium bromide (C_2) and $C_{2/}$*bis*-C_2 mixture, respectively. P-MOF-4 and P-MOF-5 were prepared by similar method of P-MOF-1, except MIL-101 was replaced by UiO-66 and ZIF-8, respectively.

Synthesis of P-COFs-X (X = 1–5). All of the COFs were prepared according to our previous work.[2–4]

P-COF-1, P-COF-2 and P-COF-3. Activated TPB-DMTP COF (1.0000 g) and *bis*-C_2 (1.0000 g) in CH_3OH (40.0 mL) were stirred at room temperature under vacuum for 24 hours. After that, AIBN (125.0 mg) in CH_3OH (5.0 mL) was added and heated to 65 ℃ under N_2 atmosphere for 24 hours. Subsequently, another CH_3OH solution (5.0 mL) of AIBN (125.0 mg) was added and heated at 65 ℃ under N_2 atmosphere for 48 hours. The final orange solid P-COF-1 was washed with CH_3OH three times and dried at 80 ℃ under vacuum for 12 hours. P-COF-2 and P-COF-3 were prepared

by the similar method, except that ionic liquid monomer was replaced by the corresponding *bis*-C_6 and *bis*-C_{12}, respectively.[2]

P-COF-4. Activated TAPB-DVA COF (100.0 mg) and *bis*-C_2 (400.0 mg) was added to CH_3OH solution (5.0 mL) under vacuum atmosphere at room temperature for 24 hours. And then, AIBN was added under N_2 atmosphere. After 48 hours of reaction, the resulting solid was washed three times with CH_3OH and dried under vacuum at 80 ℃ overnight. The brown solid product was treated with 1 M NaCl over 12 hours to yield P-COF-4.[3]

P-COF-5. A mixture of NH_2-UiO-66 (400.0 mg) and *bis*-C_2 (400.0 mg) was stirred in CH_3OH solution (20.0 mL) at room temperature under vacuum for 24 hours. Then, AIBN (50.0 mg) in CH_3OH (5.0 mL) was added and then heated to 65 ℃ under N_2 atmosphere for 24 hours. After that, additional CH_3OH solution (5.0 mL) of AIBN (50.0 mg) was added, which was heated at 65 ℃ under N_2 atmosphere for 48 hours. The resulting white solids (*bis*-PC_2@NH_2-UiO-66) were washed with CH_3OH for three times, and dried at 120 ℃ under vacuum for 12 hours. A mixture of *bis*-PC_2@NH_2-UiO-66 (40.0 mg), dimethoxyterephthaldehyde (DMTP) (93.0 mg), CH_3COOH (0.4 mL, 6 M) and n-butyl alcohol (2.0 mL) in a Pyrex tube (10.0 mL) was degassed through three freeze-pump-thaw cycles. And then, 1,3,5-tri-(4-aminophenyl)benzene (TAPB) (112.0 mg) and o-dichlorobenzene (2.0 mL) were added to the solution, which was degassed through three freeze-pump-thaw cycles. The tube was flame sealed and heated at 120 ℃ for three days. After cooling to room temperature, the precipitate was isolated by centrifugation and washed with THF for three times. Then the powder material was immersed in THF solution and dried at 120 ℃ under vacuum for 12 hours to get the as-synthesized P-COF-5.[4]

Synthesis of HOF-H_4TBAPy. Based on the previous work,[5] 10.0 mg H_4TBAPy was dissolved in 1.0 mL of DMF and 0.5 mL of 1,2,4-trichlorobenzene under ultrasonic. The mixture was heated at 80 ℃ for 48 hours to afford yellow block crystals after

cooling to room temperature. The as-synthesized HOF-H_4TBAPy washed by acetone, then dried overnight at 80 ℃ under vacuum.

Synthesis of HOF-H_4TCBP. Based on the previous work with slight modification,[6] 500.0 mg of H_4TCBP was dissolved in 5.0 mL of DMF. The mixture was slowly heated while stirring until the solid was completely dissolved. The solution was allowed to evaporate slowly at room temperature over several days. Subsequently, colorless HOF-H_4TCBP were collected and washed with acetone, then dried overnight at 80 ℃ under vacuum.

Synthesis of HOF-H_4TCPP. Based on the previous work with slight modification,[7] 50.0 mg H_4TCPP was dissolved in 22.0 mL of CH_3OH to which 3.0 mL of methanol solution of hexamine (0.02 mM) was added. The mixture heated at 60 ℃ for 5 days to afford purple crystal. The as-synthesized HOF-H_4TCPP were collected and washed with acetone, then dried overnight at 80 ℃ under vacuum.

Syntheses of G-HOFs-X (X = 1–7). In a typical procedure, 100.0 mg of activated HOF was ultrasonicated for 15 minutes. The HOF was reacted with THF/H_2O (30.0 mL/10.0 mL) solution of 200.0 mg containing grafting linker (NH_2-IL, OH-IL, and 2-(1-ethenylimidazol-4-yl)ethanol were prepared according to the reported method)[8–10] and EDC for 12 hours at room temperature. The obtained suspension was centrifugated at 8000 rpm for 20 minutes and further purified with THF and acetone several times. The resultant precipitate was collected and dried overnight at 80 ℃ under vacuum. G-HOF-1, G-HOF-2, G-HOF-3, G-HOF-4, and G-HOF-5 were synthesized by use of HOF-H_4TBAPy. G-HOF-6 and G-HOF-7 were assembled by use of HOF-H_4TCBP and HOF-H_4TCPP, respectively.

Syntheses of PG-HOFs-X (X = 1–7). The synthesis of the PG-HOFs-X was conducted based on a previously published method with slight modifications.[5] In a typical procedure, 500.0 mg of G-HOF and corresponding 2.0005 g of ionic liquids (*bis*-C_2 or *bis*-C_6) were mixed in CH_3OH under N_2 atmosphere. And then, 10.0 mL CH_3OH solution which containing AIBN (125.0 mg) was injected to initiate the

polymerization, and the mixture was heated to 70 ℃ under N_2 for 24 hours. The same amount of AIBN/CH_3OH solution was injected and the reaction was kept at 70 ℃ for another 24 hours. The as-synthesized powder was collected and washed with CH_3OH, then dried overnight at 80 ℃ under vacuum. The PG-HOFs-X were obtained after soaking the products with 2 M HCl for two days.

Synthesis of PG-HOF-2/PAA beads. In a typical procedure, 800.0 mg of PG-HOF-2 powder was dispersed in 15.0 mL of sodium alginate (SA) aqueous solution. Meanwhile, 1.1504 g of $CaCl_2$ was dissolved in 200.0 mL of polyacrylic acid (PAA, M_w = 2000) aqueous solution, which served as the curing solution. Subsequently, the PG-HOF-2/SA suspension was added into the curing solution using a syringe. The resultant spherical PG-HOF-2/PAA beads were collected and thoroughly washed with water, and then activated upon heating to 80 ℃ under vacuum. The approaches of other CPFs/PAA were similar to that of PG-HOF-2/PAA, except that CPFs was replaced by different P-MOFs, P-COFs, and PG-HOFs.

Synthesis of PG-HOF-2/PES beads. In a typical procedure, 1.0012 g of polyether sulfone (PES) was fully dissolved in 10.0 mL of DMF through mechanical stirring for 24 hours at 60 ℃, yielding a transparent PES solution with uniform viscosity. Add 800.0 mg of PG-HOF-2 powder to the PES solution under stirring overnight. And then, the suspension was added dropwise by syringe into a precooled water/ethanol (v/v = 1:1) solution. PG-HOF-2/PES beads were immediately formed by solvent/water exchange. The resultant beads were collected and thoroughly washed with water and ethanol, and then activated upon heating to 80 ℃ under vacuum. The approaches of other CPFs/PES were similar to that of PG-HOF-2/PES, except that CPFs was replaced by different P-MOFs, P-COFs, and PG-HOFs.

4. Batch sorption experiments

Due to the radiological operation limits of ^{99}Tc, ReO_4^- was used as a surrogate for $^{99}TcO_4^-$, because they have almost identical charge densities and chemical properties.

The concentration of ReO_4^- remaining in the water phase was determined by ICP-MS. All the adsorption studies have been carried out taking following respective concentration of different anionic analytes in pure aqueous solution. In case of stock solution of oxyanions, potassium perrhenate ($KReO_4$), the initial concentration was 25 ppm.

General equations.

$$\text{Removal percentage (\%)} = \frac{c_0 - c_e}{c_0} \times 100\% \quad (1)$$

$$q_e = \frac{c_0 - c_e}{m} \times V \quad (2)$$

$$k_d = \frac{c_0 - c_e}{c_e} \times \frac{V}{m} \quad (3)$$

where q_e (mg g^{-1}) is sorption capacity at equilibrium, K_d (mL g^{-1}) is distribution coefficient, c_0 (mg g^{-1}) and c_e (mg g^{-1}) are the initial and equilibrium concentrations of adsorbate, V (mL) is the volume of solution, and m (g) is the mass of sorbent.

To study the removal thermodynamics, Langmuir and Freundlich sorption models were used.

The equation of Langmuir model is

$$\frac{c_e}{q_e} = \frac{1}{q_m k_L} + \frac{c_e}{q_m} \quad (4)$$

where c_e is the equilibrium concentration of Re (mg g^{-1}), q_e and q_m are the equilibrium and maximum Re sorption amount (mg g^{-1}). k_L is a constant indirectly related to the sorption amount and sorption energy (L mg^{-1}), characterizing the affinity of ReO_4^- with the sorbent. The fitting line was obtained by plotting c_e/q_e against c_e, and q_m and k_L could be calculated from the slope and intercept.

The equation of the Freundlich model is

$$\ln q_e = \ln k_F + \frac{1}{n} \ln c_e \quad (5)$$

where c_e and q_e are the equilibrium concentration of Re in ReO_4^- (mg g^{-1}) and equilibrium Re sorption amount (mg g^{-1}), and k_F and n are the Freundlich constants

related to sorption amount and sorption intensity, characterizing the affinity of ReO_4^- with the sorbent.

ReO_4^- Measurements. In a typical batch experiment, 10.0 mg of sorbent material was added to 5.0 mL ReO_4^- water solution at a given concentration at room temperature. The reaction system was adjusted to the desired pH value with NaOH or HNO_3 solution. The sample was separated at different time intervals. The concentration of Re remaining in the water phase was determined by ICP-MS.

Sorption isotherm experiments. 10.0 mg of sorbent material (PG-HOF-2, PG-HOF-2/PAA, and PG-HOF-2/PES) was added to a water solution (5.0 mL) of $KReO_4$ at a given concentration (50–800 ppm Re, pH = 7). The samples were placed in solution for 12 hours to ensure the equilibrium.

ReO_4^- sorption data fitting by kinetics models. 10.0 mg of sorbent (PG-HOF-2, PG-HOF-2/PAA, and PG-HOF-2/PES) was added into a $KReO_4$ water solution (5.0 mL, 25 ppm Re). The samples were collected after adding for 30 s, 1 min, 2 min, 5 min, 10 min, and 30 min, respectively. The concentration of Re remaining in water phase was determined using ICP-MS. The equation of linearized form of pseudo-second-order model is

$$\frac{t}{q_t} = \frac{t}{q_e} + \frac{1}{k_2 q_e^2} \quad (6)$$

in which q_t is the uptake amount of Re at t min (mg g^{-1}), q_e is the uptake amount of Re at equilibrium (mg g^{-1}), and k_2 is the pseudo-second-order rate constant (g mg^{-1} min^{-1}). The model parameter and correlation coefficient are listed in Table S2.

Effect of pH value. The effect of pH value on ReO_4^- sorption was studied in solutions of pH = 1–11 (regulated by HNO_3 or NaOH). 10.0 mg of sorbent material (PG-HOF-2, PG-HOF-2/PAA and PG-HOF-2/PES) was added into a $KReO_4$ water solution (5.0 mL, 25 ppm Re). The sample was placed for 12 hours to ensure the equilibrium. The concentration of Re remaining in the water phase was determined by ICP-MS.

Anion exchange selectivity. The competing effect of other anion (NO_3^-, NO_2^-, ClO_4^-, SO_4^{2-}, PO_4^{3-}, and CO_3^{2-}) was initially performed by adding 0.5 mM $NaNO_3$, $NaNO_2$, $NaClO_4$, Na_2SO_4, K_3PO_4, and Na_2CO_3 solution, receptively, to a 0.5 mM $KReO_4$ solution. After that, 10.0 mg of sorbent material (PG-HOF-2, PG-HOF-2/PAA, and PG-HOF-2/PES) was added in the above solution (5.0 mL), respectively. The Re concentrations in the initial solution and filtrate were determined by ICP-MS.

Selectivity of excessive competing NO_3^- and SO_4^{2-}. The competition effect of NO_3^- was performed by adding 1.50, 7.50, 15.00, 30.00, 60.00 or 90.00 mM $NaNO_3$ solutions, respectively, into a 0.15 mM ReO_4^- solution. Similarly, adding 0.80, 4.00, 8.00, 16.00, 32.00 or 48.00 mM Na_2SO_4 solutions respectively into a 0.08 mM ReO_4^- solution, 10.0 mg of PG-HOF-2/PES sorbent material was added into mixed solution (5 mL) for 12 hours. The Re concentration in the initial solution and filtrate were determined by ICP-MS.

Reusability. The initial concentration of $KReO_4$ solution (pH = 7, 25 ppm Re) used in the recycle test was constant. Sorption was carried out at a solid/liquid ratio of 2 g L^{-1}. Taking the first cycle as an example, PG-HOF-2@Re powder, PG-HOF-2/PAA@Re beads, and PG-HOF-2/PES@Re beads were immersed in 3 M HCl for one hours, respectively, then washed by deionized water and collected. After drying at 80 ℃ for 2 hours under vacuum, the regenerated material could be prepared for the next adsorption.

Dynamic sorption experiment for tap water containing 20 ppm Re. The dynamic sorption system of PG-HOF-2/PAA beads was composed of two sequential polypropylene columns (ID: 12.8 mm, L: 60.0 mm). PG-HOF-2/PAA beads (1.0021 g) were selected as the filler of each column. The flow rate of the gas sparging system was controlled by a flowmeter to achieve different two-phase flow regimes. A feed solution of 20 ppm Re contaminating tap water with a flow rate of 1.0 mL min^{-1} was used. Meanwhile, the other dynamic experiment was performed with PG-HOF-2/PES (1.0022 g) and quartz sand mixed in columns, with the help of peristaltic pump at the

same flow condition. The performance of the PG-HOF-2/PAA and PG-HOF-2/PES beads in the sorption system was determined by values of Re concentration at the outlet. All the samples were collected at the outlet of the second column and characterized by ICP-MS.

Sorption in simulated waste system. The simulated Hanford LAW melter recycle stream were prepared (Table S8), where $^{99}TcO_4^-$ was replaced by ReO_4^-. Purolite A530E, PG-HOF-2, PG-HOF-2/PAA, and PG-HOF-2/PES (100.0 mg) was added into 5.0 mL of LAW for 12 hours, respectively. The residual Re concentration in water phase was determined by ICP-MS.

Dynamic experiment system for sorption of pre-treated LAW solution. Equipped with peristaltic pump, LAW solution was flow through the Purolite A530E resin (1.0002 g) with the flow rate of 1.0 mL min^{-1}. The resin was pretreated by first soaking in water and filtration, then washed with water until the pH of the eluate was near neutral. The effluent was collected at the outlet as the pre-treated LAW solution and characterized by ICP-MS. Pre-treated LAW solutions (12.298 ppb Re) were used as the feed solution to pass through the column containing Purolite A530E resin (1.0007 g), PG-HOF-2/PAA (1.0010 g) and PG-HOF/PES (1.0020 g) with the flow rate of 1.0 mL min^{-1}, respectively. Adsorption performance of the PG-HOFs beads at extremely low concentrations was determined by values of Re concentration at the outlet. After each cycle, the packed columns were flushed by 3 M HCl at 2.0 mL min^{-1}. Then the PG-HOF-2/PES beads were dried in the column by air flow at room temperature. The samples were collected at the outlet and characterized by ICP-MS.

Removal ability of CPFs powder and CPFs/PES beads for pre-treated LAW solution. 10.0 mg of sorbent material (CPFs powder and CPFs/PES beads) was added to a pre-treated LAW solution (5.0 mL). The samples were placed in solution for 12 hours to ensure the equilibrium.

5. Computational method

To better understand the excellent adsorption properties of PG-HOF-2/PES toward TcO_4^-/ReO_4^-, the positively charged local structures of the as-developed PG-HOF-2 and the commercial resin Purolite A530E were modeled by the A $[CH_3\text{-}C_3N_2H_3\text{-}C_2H_4\text{-}O_2C\text{-}CH_3]^{2+}$ fragment, A' $[C_3N_2H_3\text{-}C_2H_4\text{-}C_3N_2H_3]^{2+}$ fragment and the B $[C_6H_5\text{-}CH_2\text{-}R_4N]^+$ fragment, respectively. Geometry optimizations were performed with the M062X functional with D3 version of Grimme's dispersion corrections.[11,12] The def2-SVP basis set was employed for all atoms.[13] All of the calculations were performed with Gaussian 09.[14] The quantitative analysis of electrostatic potential on van der Waals (vdW) surface was performed using the Multiwfn 3.8 program.[15] Additionally, single point energy calculations were performed with the M062X-D3 functional, and the def2-TZVP for all atoms,[16] with the inclusion of solvation energy corrections based on SMD implicit solvent model with water as solvent (ε = 78.3553).[17] All the reported enthalpy values are the sum of the electronic energy from the single point calculations and the thermal correction to enthalpy obtained by the frequency calculations.

Section S2. Characterization Data

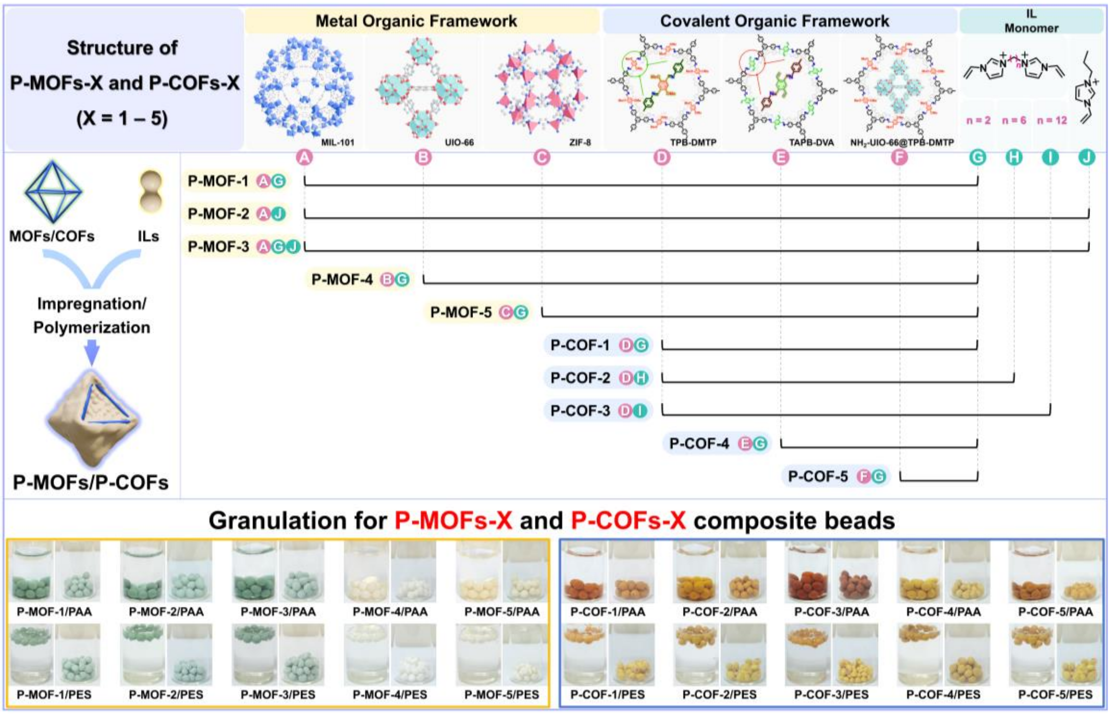

Figure S1. Schematic diagrams for fabrication of P-MOFs and P-COFs composite beads, as well as digital photographs of twenty wet and dry P-MOFs and P-COFs composite beads.

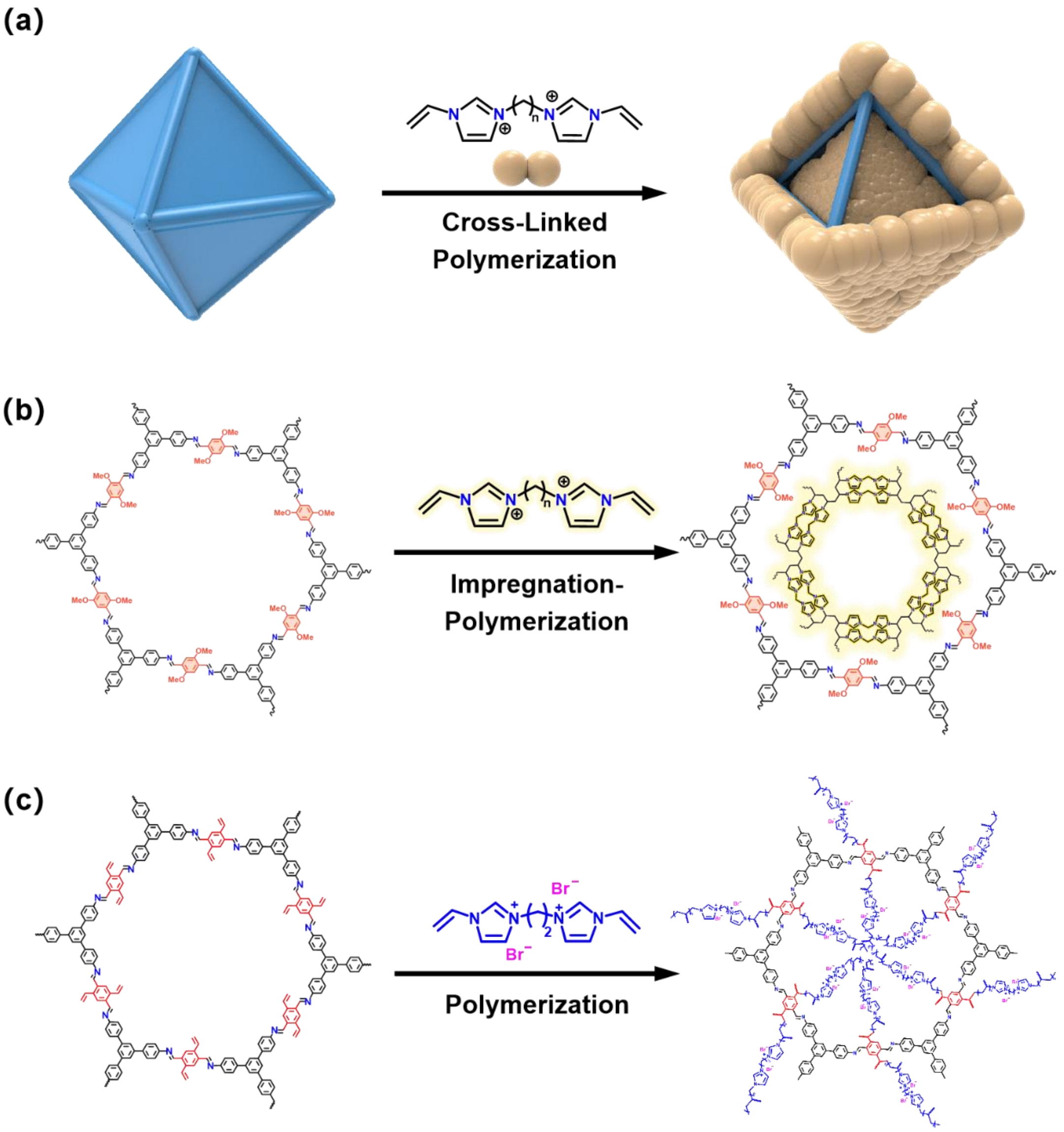

Figure S2. Detailed synthetic strategy of P-MOFs and P-COFs. (a) Schematic diagram of P-MOF-X (X = 1–5) and P-COF-5. (b) The synthetic route of P-COF-X (X = 1–3). (c) The synthetic route of P-COF-4.

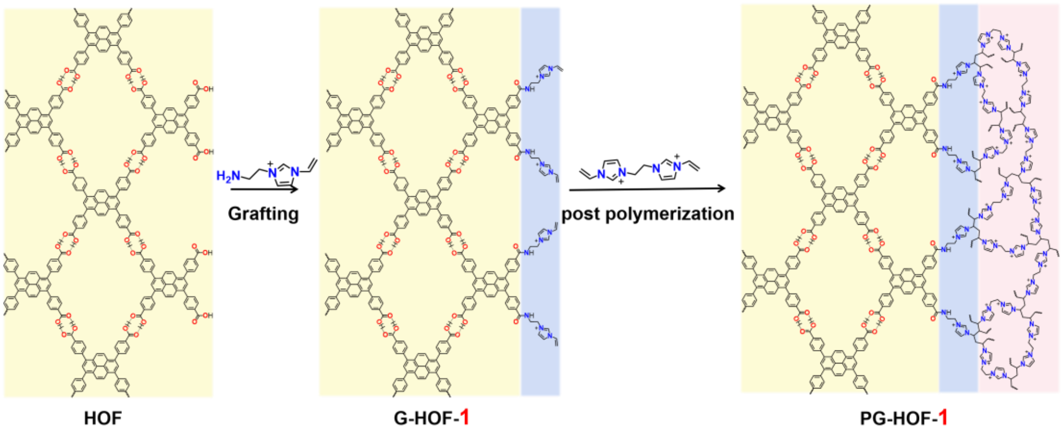

(a)

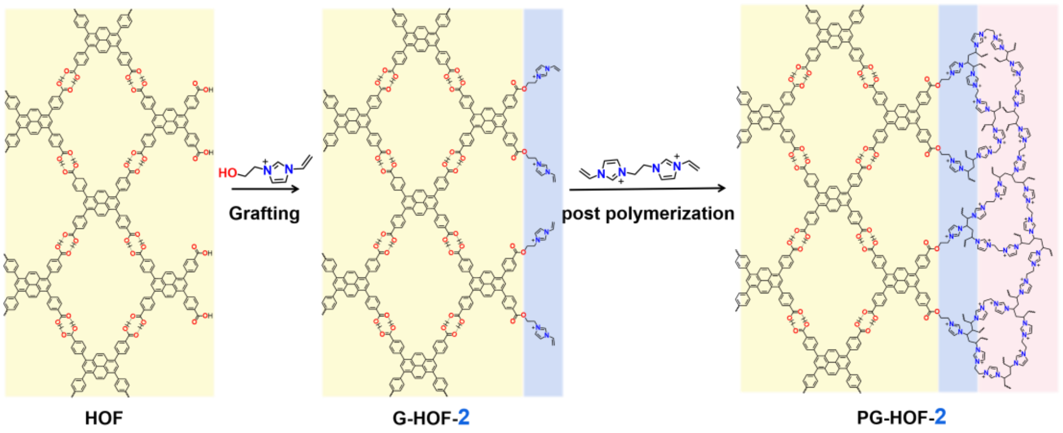

(b)

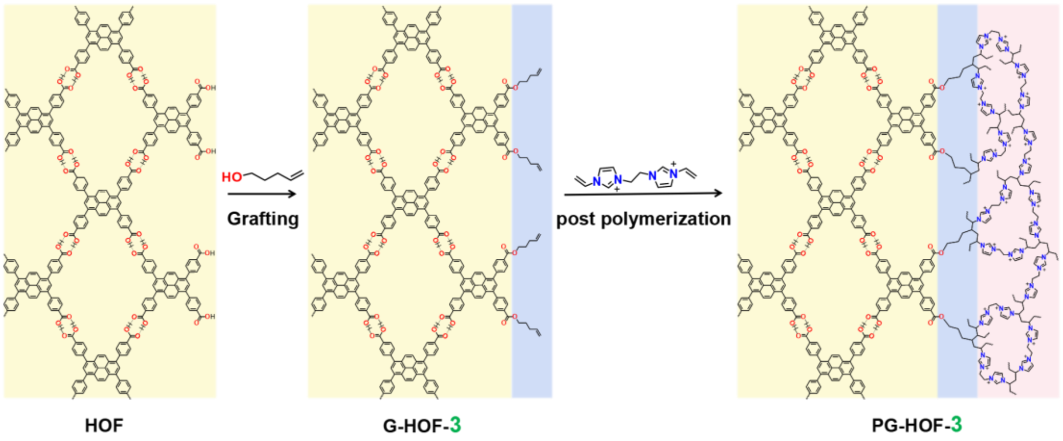

(c)

Figure S3. Synthetic routes of (a) PG-HOF-1, (b) PG-HOF-2, and (c) PG-HOF-3.

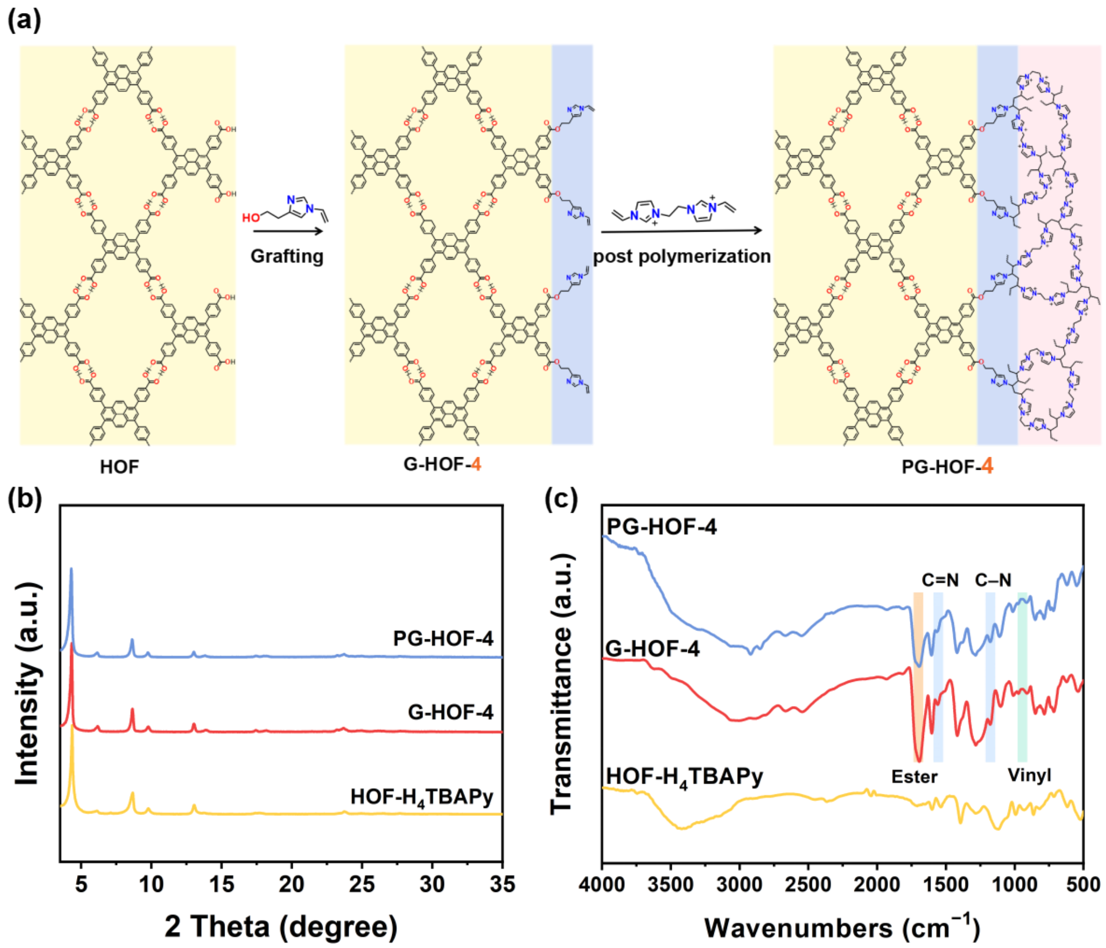

Figure S4. (a) Synthetic route of PG-HOF-4. (b) PXRD patterns and (c) FT-IR spectra of HOF-H_4TBAPy, G-HOF-4, and PG-HOF-4.

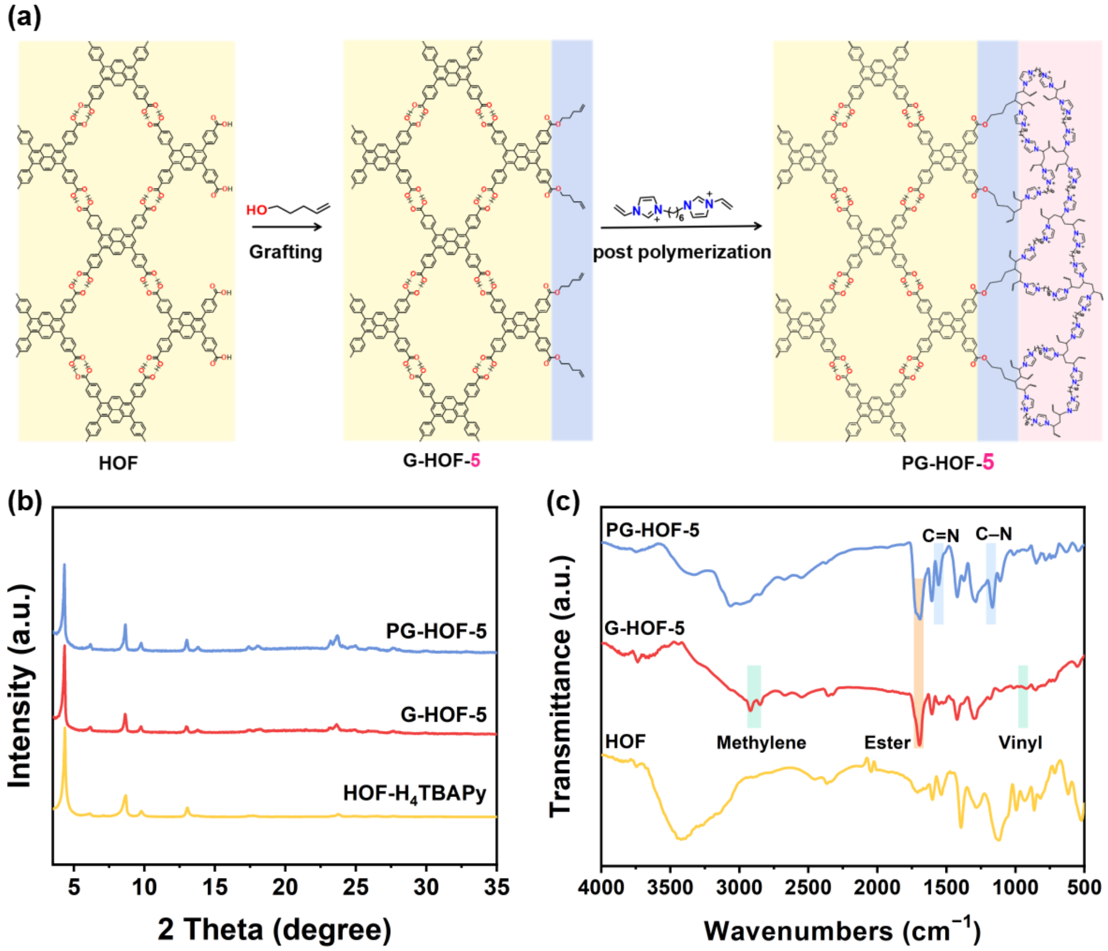

Figure S5. (a) Synthetic route of PG-HOF-5. (b) PXRD patterns and (c) FT-IR spectra of HOF-H_4TBAPy, G-HOF-5, and PG-HOF-5.

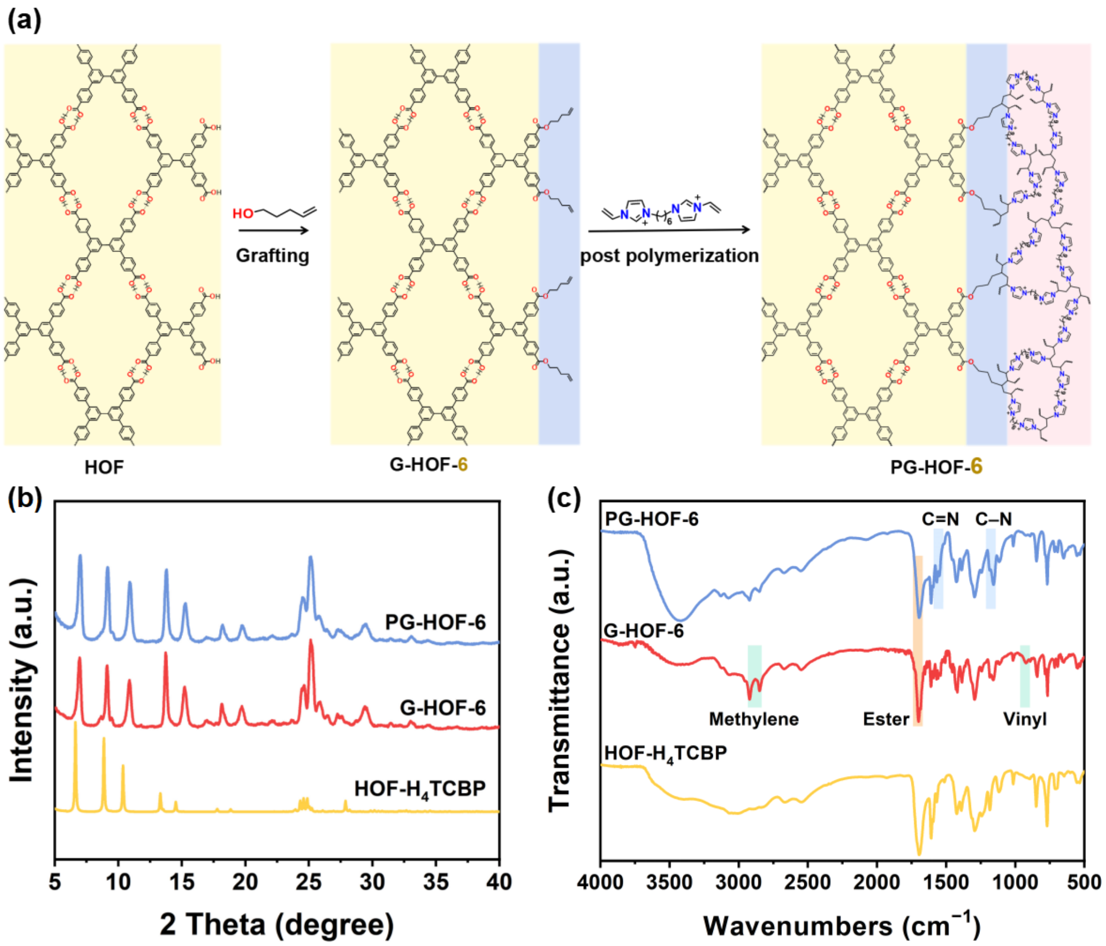

Figure S6. (a) Synthetic route of PG-HOF-6. (b) PXRD patterns and (c) FT-IR spectra of HOF-H_4TCBP, G-HOF-6, and PG-HOF-6.

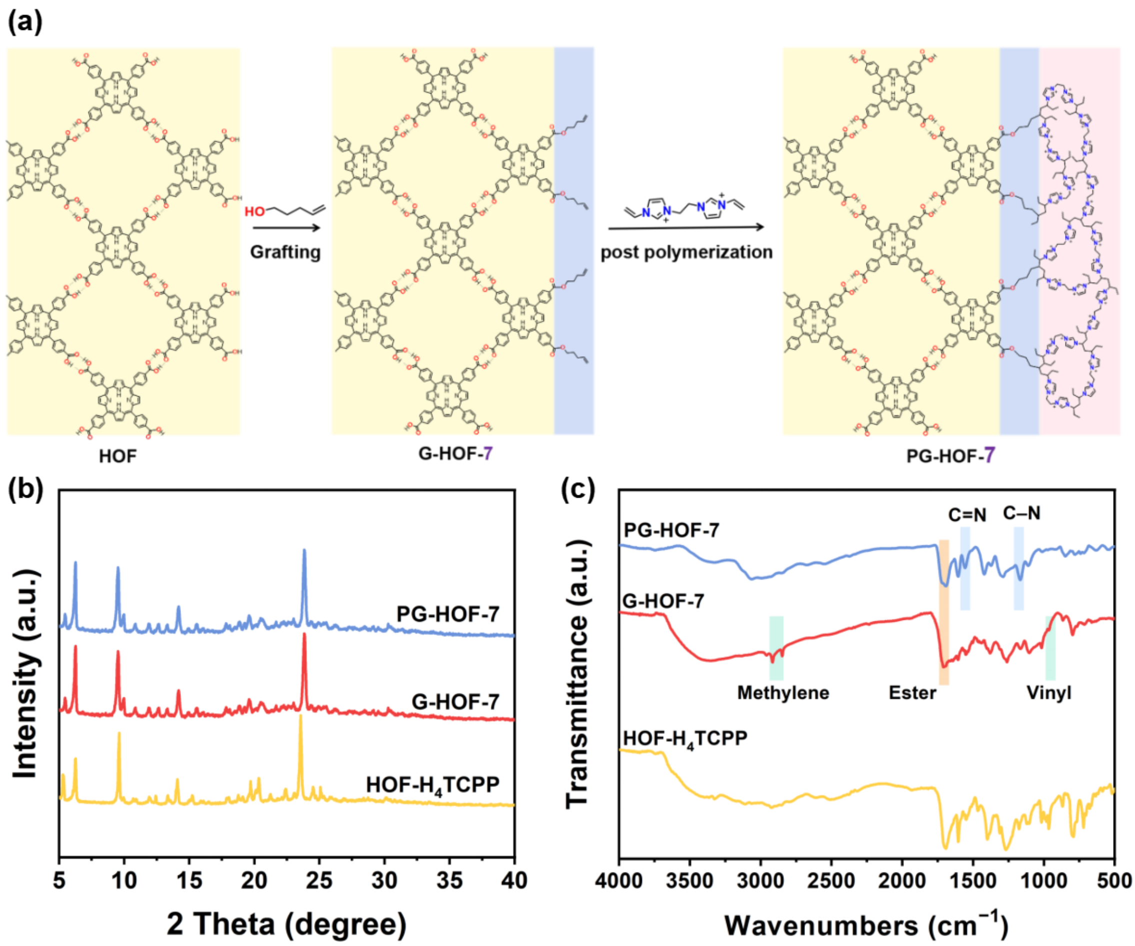

Figure S7. (a) Synthetic route of PG-HOF-7. (b) PXRD patterns and (c) FT-IR spectra of HOF-H_4TCPP, G-HOF-7, and PG-HOF-7.

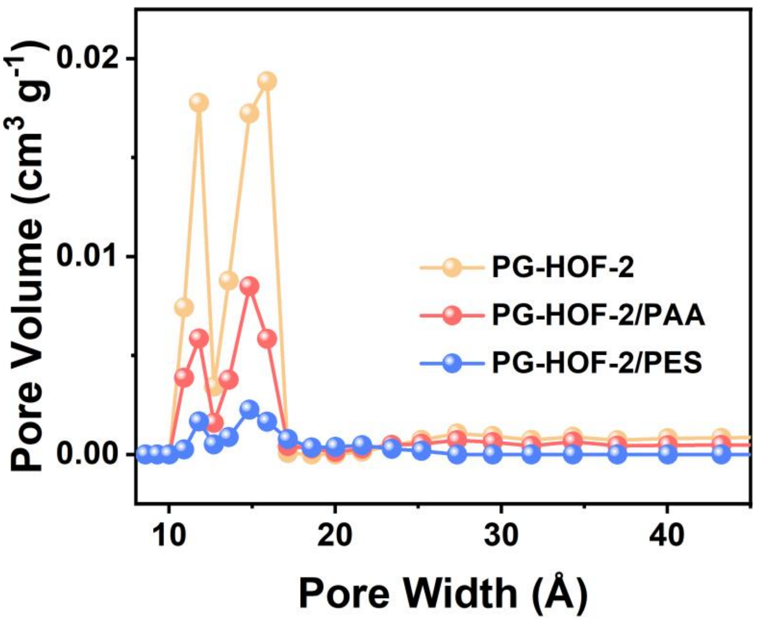

Figure S8. Pore size distribution of PG-HOF-2, PG-HOF-2/PAA, and PG-HOF-2/PES.

序号	时间<br>min	峰名称	峰类型	峰面积<br>μS*min	峰高<br>μS	样品量<br>mg/Kg
1	6.43	Cl	BMB*	6.531	38.988	21404.0418
		总计：		6.53	38.99	21404.04

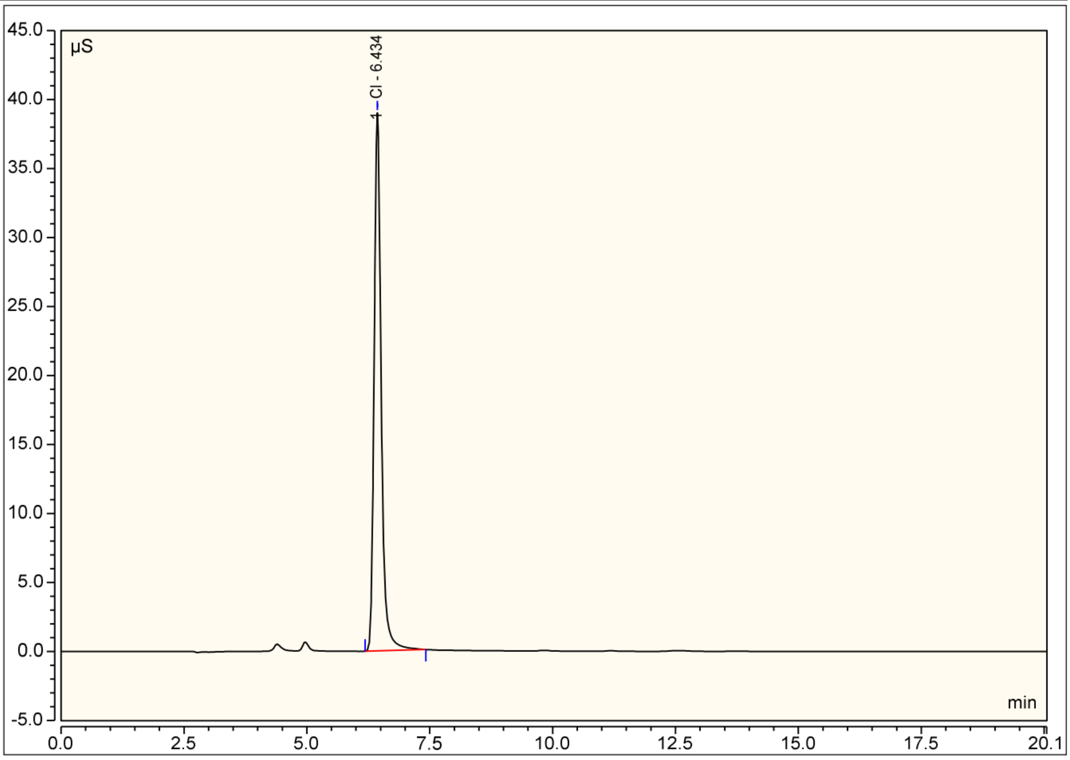

Figure S9. Ion Chromatography spectrum for Cl element analysis. The content of Cl^{-} within PG-HOF-2 is approximately 0.21 wt%.

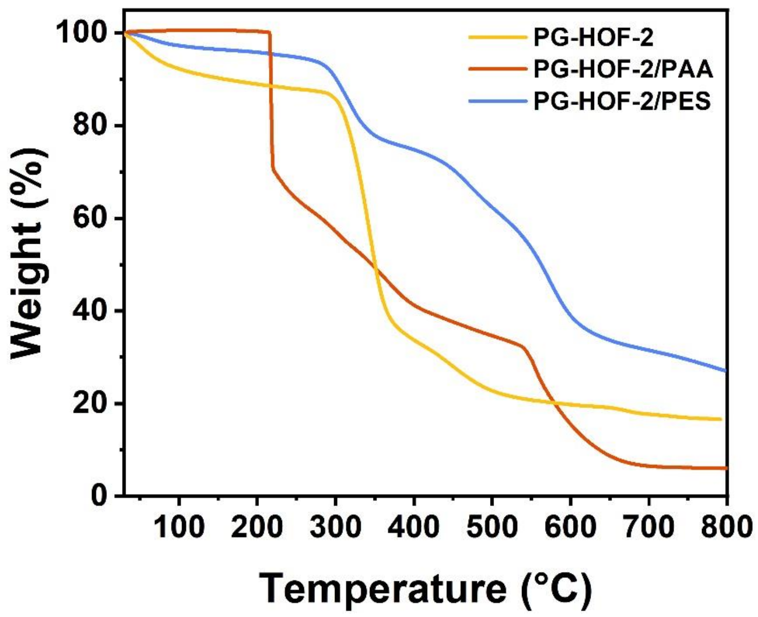

Figure S10. TGA analysis of the PG-HOF-2, PG-HOF-2/PAA, and PG-HOF-2/PES.

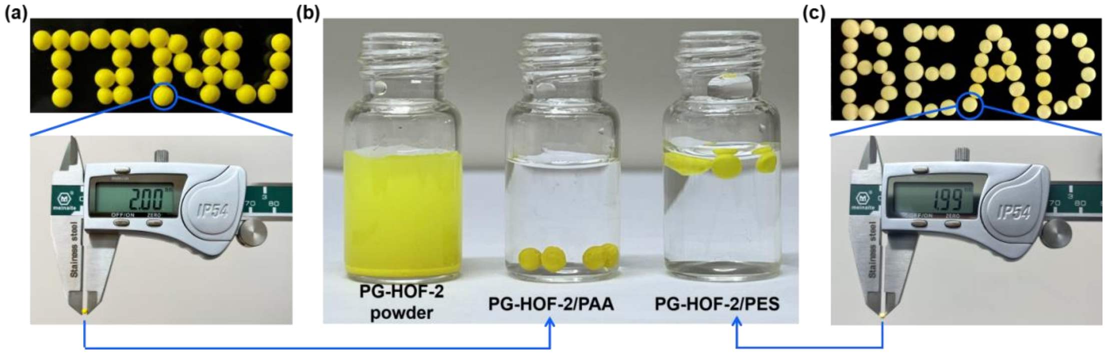

Figure S11. Diagram of PG-HOF-2 composite beads. (a) Optical image of dried PG-HOF-2/PAA beads on a flat surface. (b) Photographs of the PG-HOF-2 powders, PG-HOF-2/PAA beads, and PG-HOF-2/PES beads immersed in water solution. (c) Optical image of dried PG-HOF-2/PES beads on a flat surface. The diameter of dried composite beads was both 2.00 ± 0.1 mm.

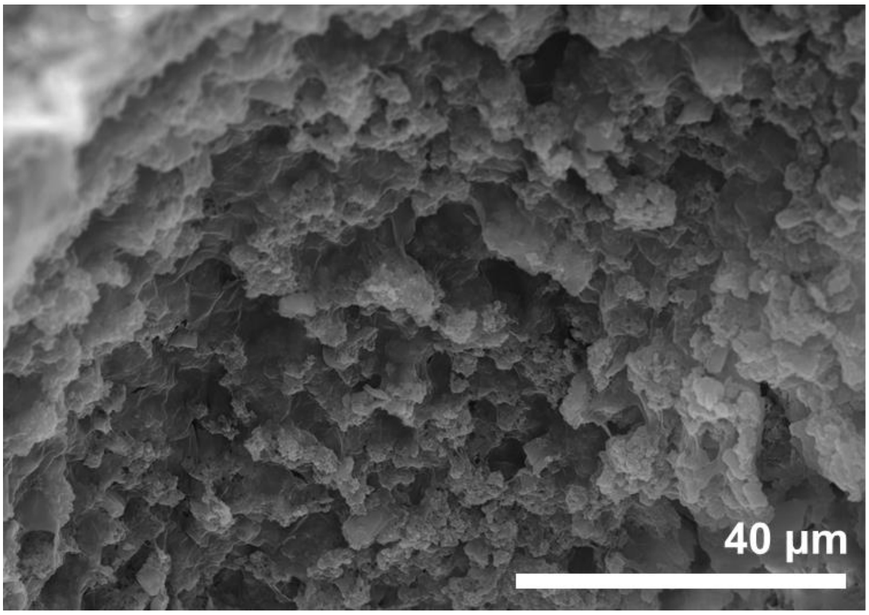

Figure S12. Enlarged SEM image of the inside morphology of PG-HOF-2/PAA bead.

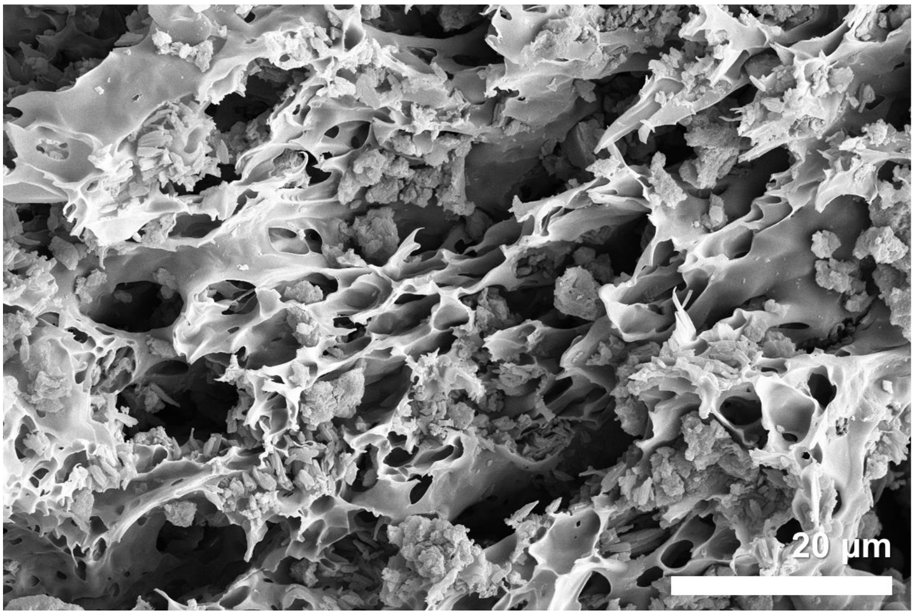

Figure S13. Enlarged SEM image of the inside morphology of PG-HOF-2/PES bead.

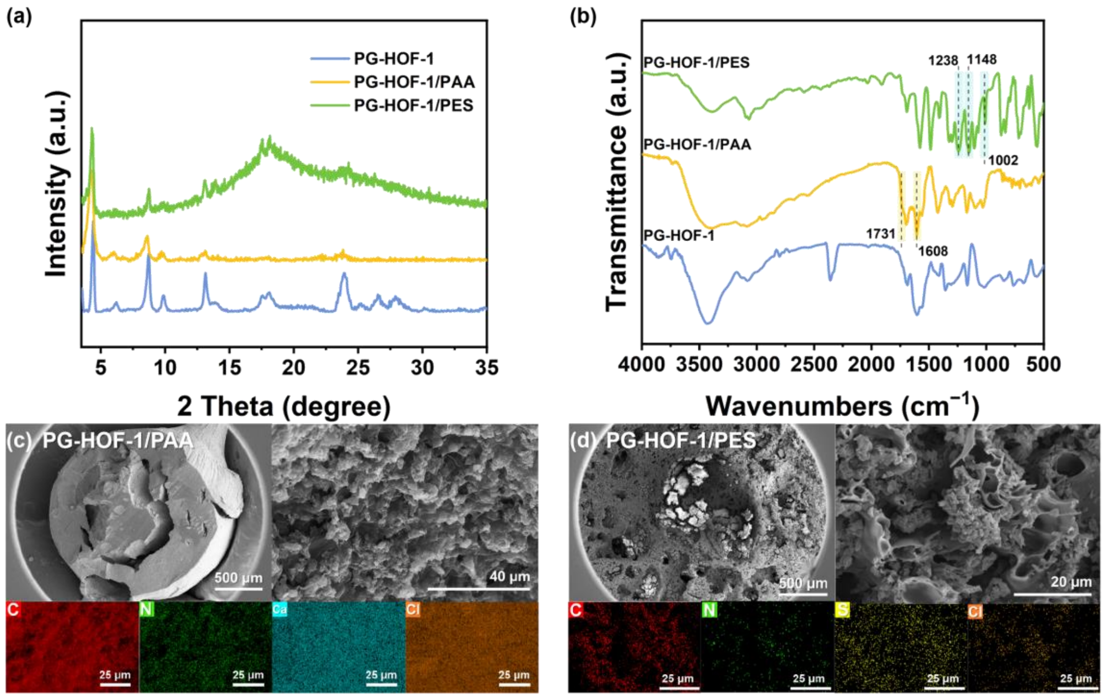

Figure S14. Characterizations of PG-HOF-1 composite beads. (a) PXRD pattern and (b) FT-IR spectra of pristine PG-HOF-1 powder, PG-HOF-1/PAA bead, and PG-HOF-1/PES bead. SEM image of the whole intersection and the closeup (including elemental mapping analysis) of (c) PG-HOF-1/PAA bead and (d) PG-HOF-1/PES bead.

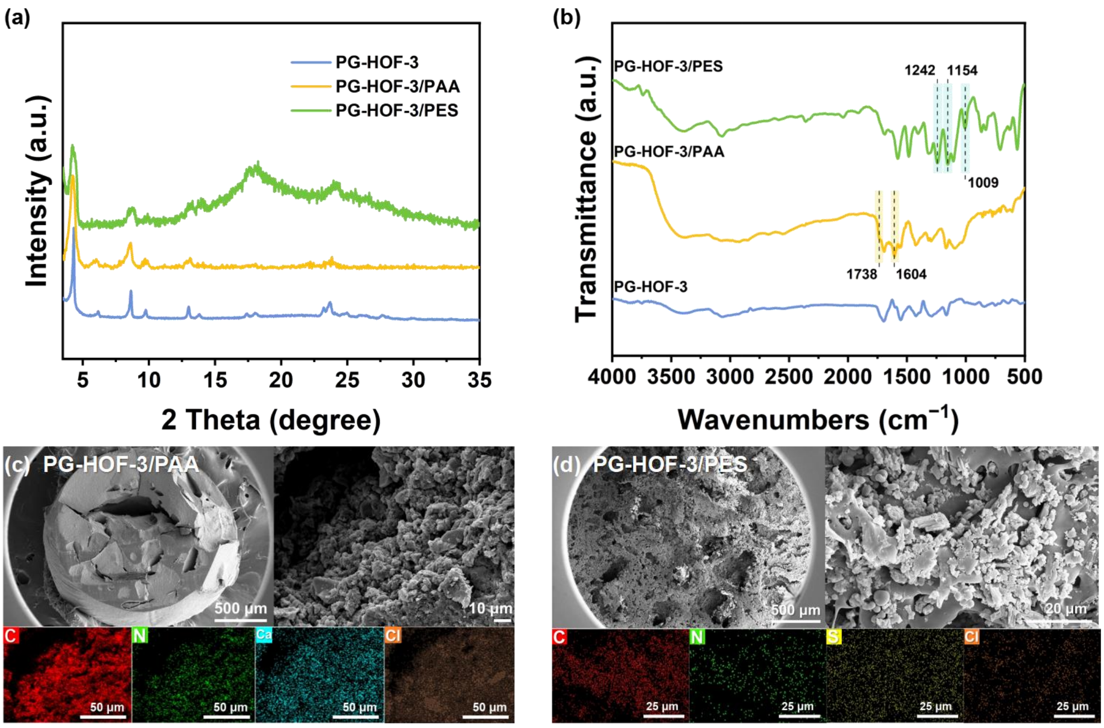

Figure S15. Characterizations of PG-HOF-3 composite beads. (a) PXRD pattern and (b) FT-IR spectra of pristine PG-HOF-3 powder, PG-HOF-3/PAA bead, and PG-HOF-3/PES bead. SEM image of the whole intersection and the closeup (including elemental mapping analysis) of (c) PG-HOF-3/PAA bead and (d) PG-HOF-3/PES bead.

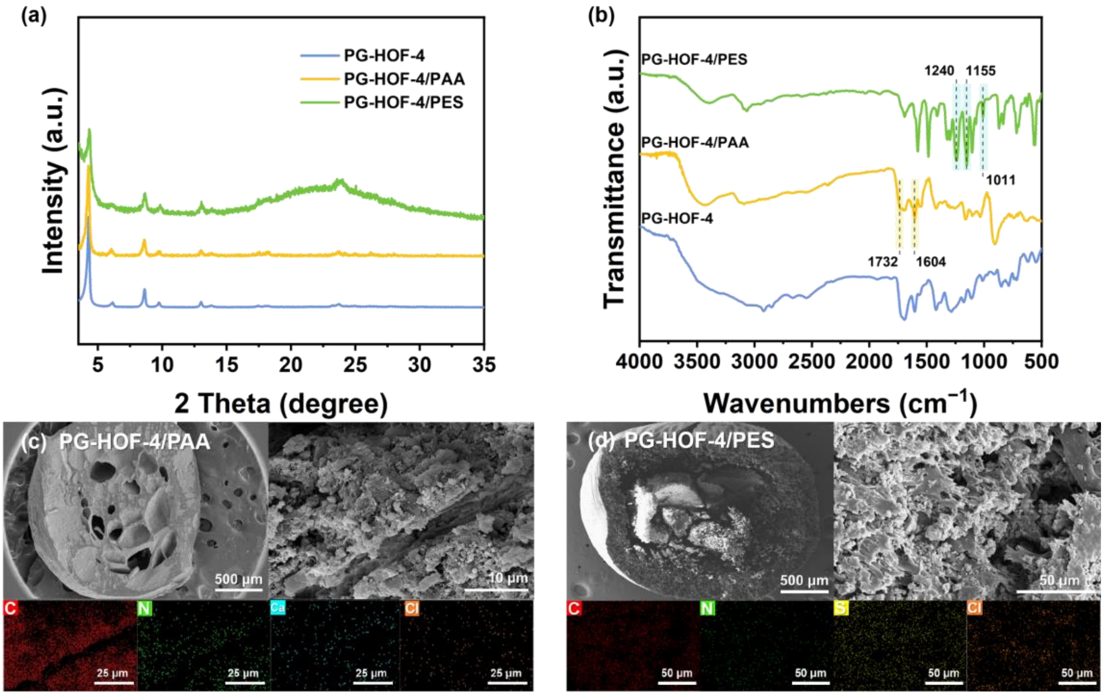

Figure S16. Characterizations of PG-HOF-4 composite beads. (a) PXRD pattern and (b) FT-IR spectra of pristine PG-HOF-4 powder, PG-HOF-4/PAA bead, and PG-HOF-4/PES bead. SEM image of the whole intersection and the closeup (including elemental mapping analysis) of (c) PG-HOF-4/PAA bead and (d) PG-HOF-4/PES bead.

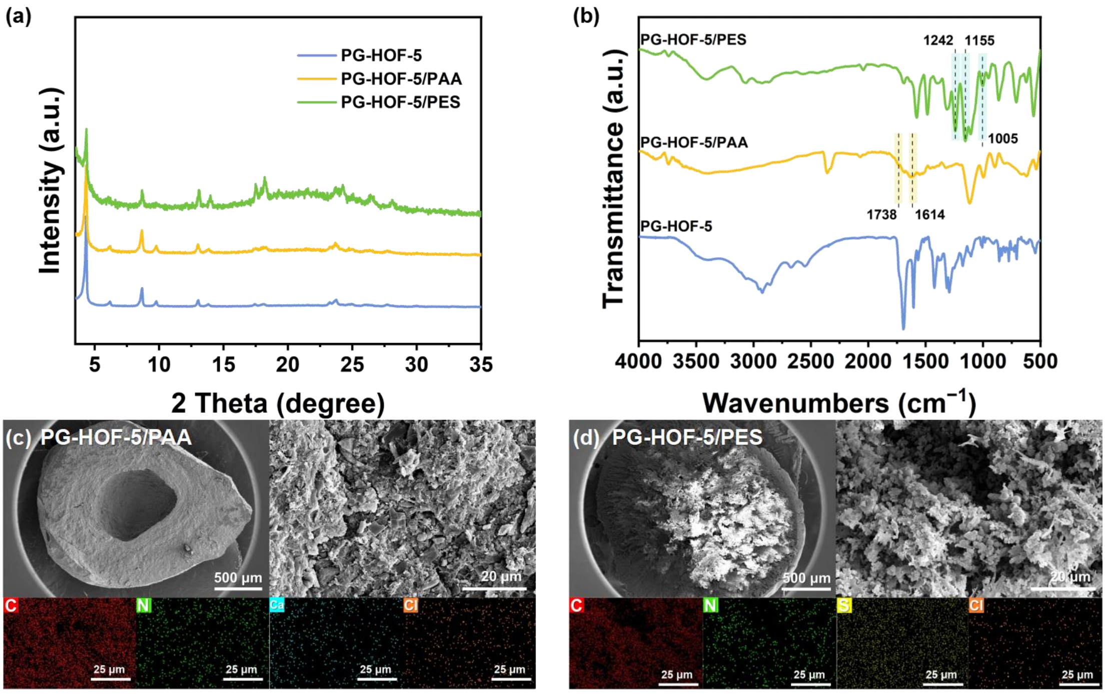

Figure S17. Characterizations of PG-HOF-5 composite beads. (a) PXRD pattern and (b) FT-IR spectra of pristine PG-HOF-5 powder, PG-HOF-5/PAA bead, and PG-HOF-5/PES bead. SEM image of the whole intersection and the closeup (including elemental mapping analysis) of (c) PG-HOF-5/PAA bead and (d) PG-HOF-5/PES bead.

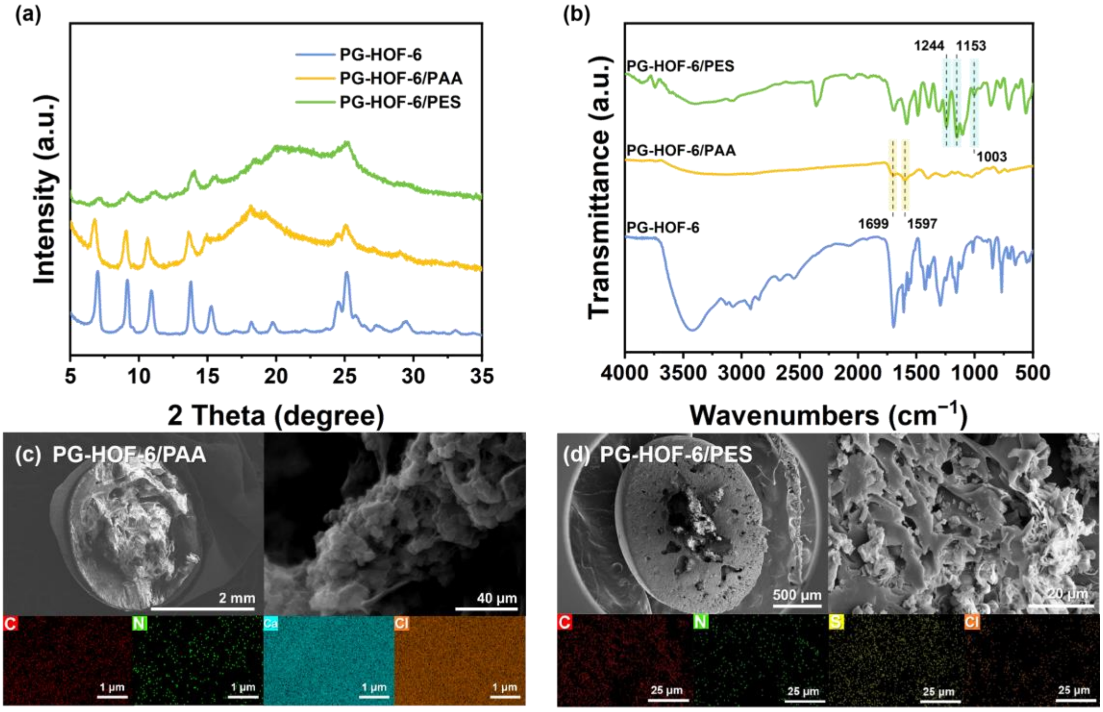

Figure S18. Characterizations of PG-HOF-6 composite beads. (a) PXRD pattern and (b) FT-IR spectra of pristine PG-HOF-6 powder, PG-HOF-6/PAA bead, and PG-HOF-6/PES bead. SEM image of the whole intersection and the closeup (including elemental mapping analysis) of (c) PG-HOF-6/PAA bead and (d) PG-HOF-6/PES bead.

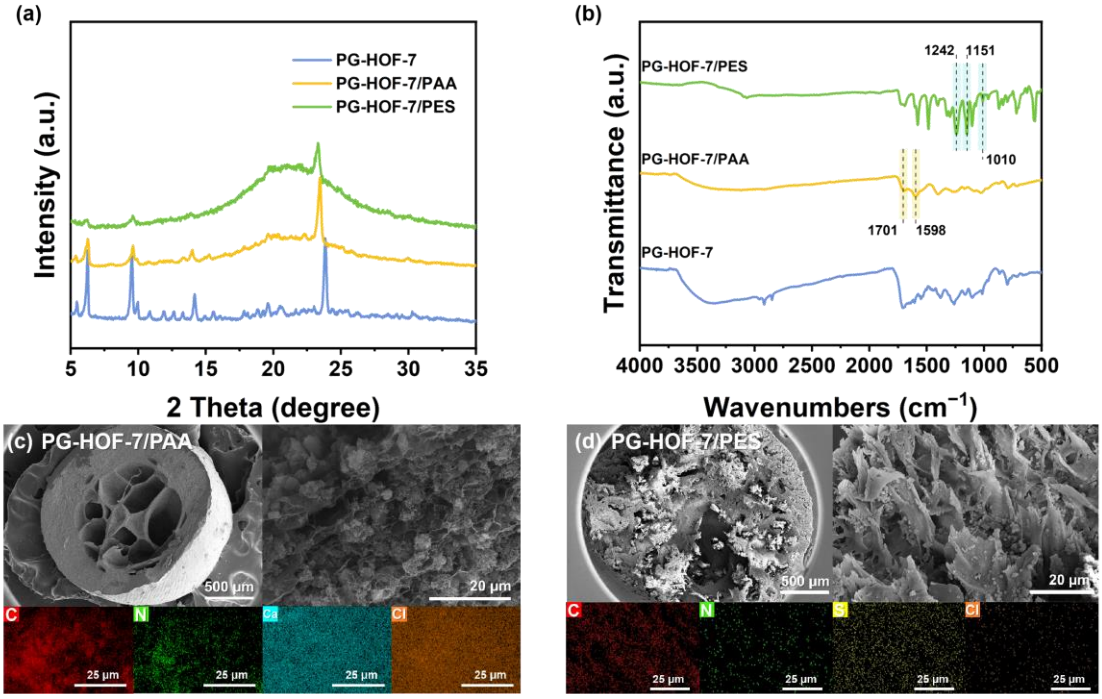

Figure S19. Characterizations of PG-HOF-7 composite beads. (a) PXRD pattern and (b) FT-IR spectra of pristine PG-HOF-7 powder, PG-HOF-7/PAA bead, and PG-HOF-7/PES bead. SEM image of the whole intersection and the closeup (including elemental mapping analysis) of (c) PG-HOF-7/PAA bead and (d) PG-HOF-7/PES bead.

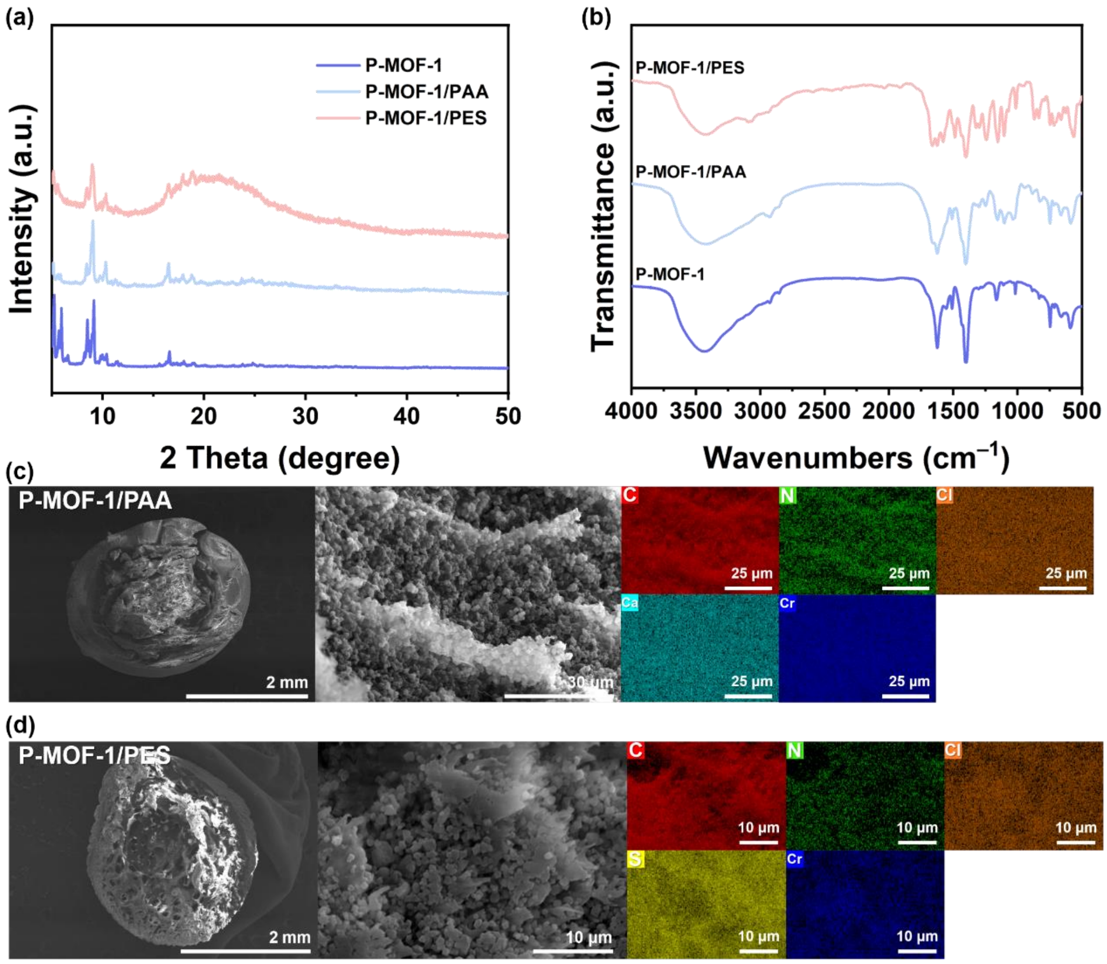

Figure S20. Characterizations of P-MOF-1 composite beads. (a) PXRD pattern and (b) FT-IR spectra of pristine P-MOF-1 powder, P-MOF-1/PAA bead, and P-MOF-1/PES bead. SEM image of the whole intersection and the closeup (including elemental mapping analysis) of (c) P-MOF-1/PAA bead and (d) P-MOF-1/PES bead.

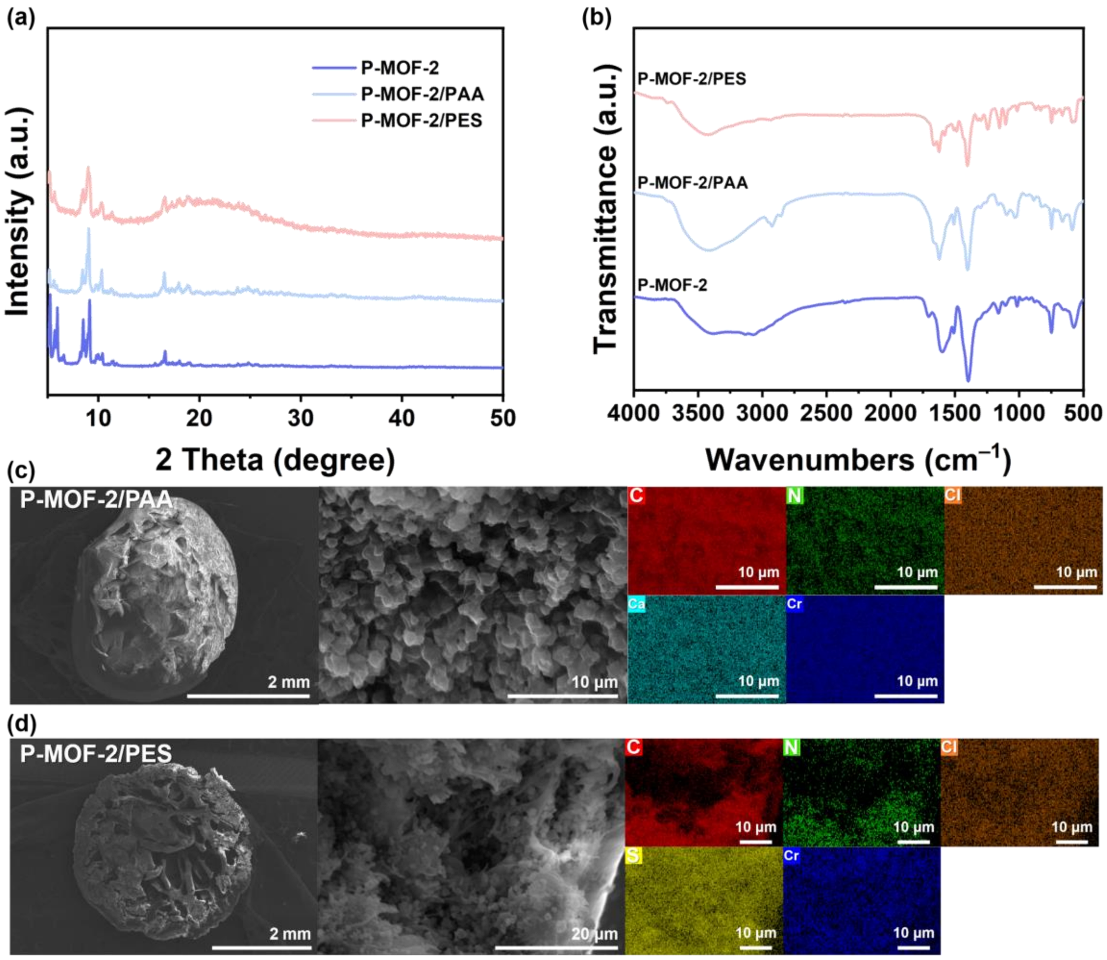

Figure S21. Characterizations of P-MOF-2 composite beads. (a) PXRD pattern and (b) FT-IR spectra of pristine P-MOF-2 powder, P-MOF-2/PAA bead, and P-MOF-2/PES bead. SEM image of the whole intersection and the closeup (including elemental mapping analysis) of (c) P-MOF-2/PAA bead and (d) P-MOF-2/PES bead.

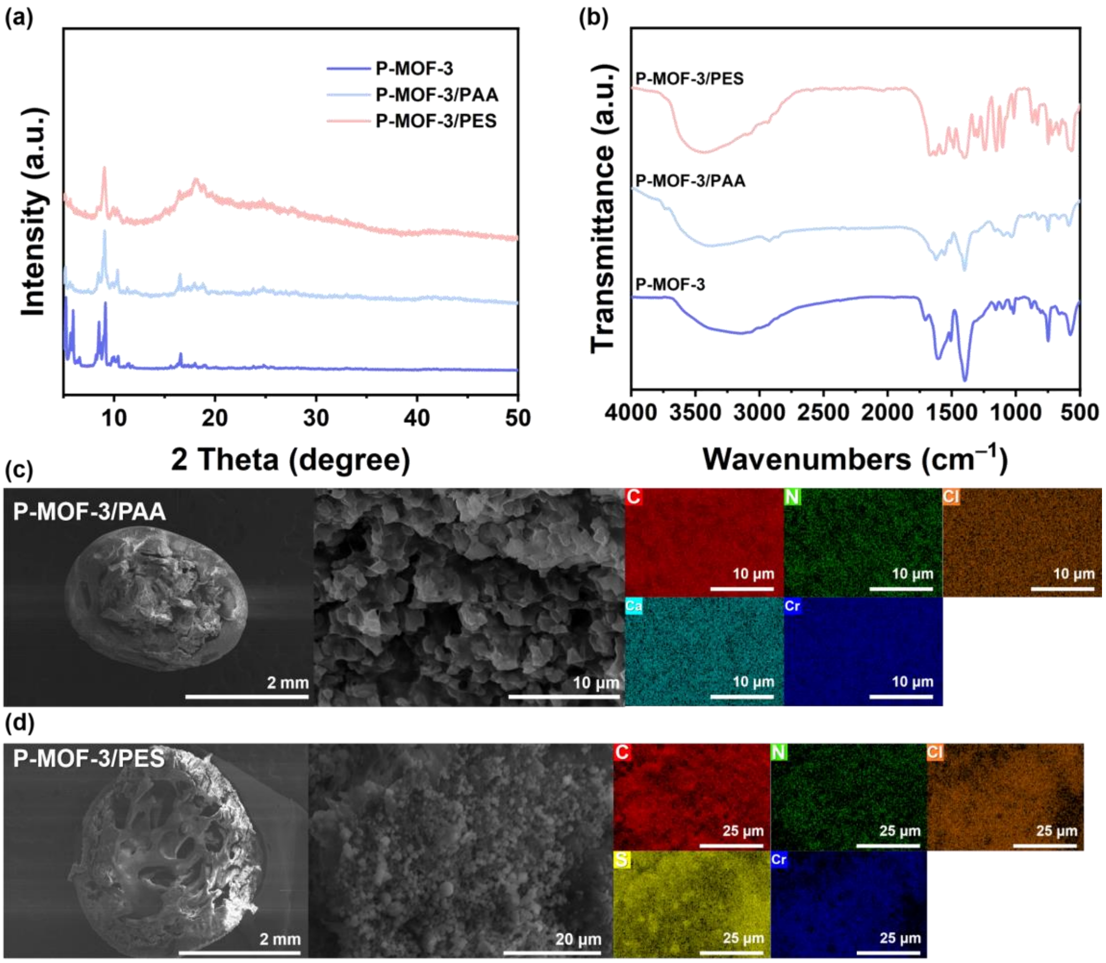

Figure S22. Characterizations of P-MOF-3 composite beads. (a) PXRD pattern and (b) FT-IR spectra of pristine P-MOF-3 powder, P-MOF-3/PAA bead, and P-MOF-3/PES bead. SEM image of the whole intersection and the closeup (including elemental mapping analysis) of (c) P-MOF-3/PAA bead and (d) P-MOF-3/PES bead.

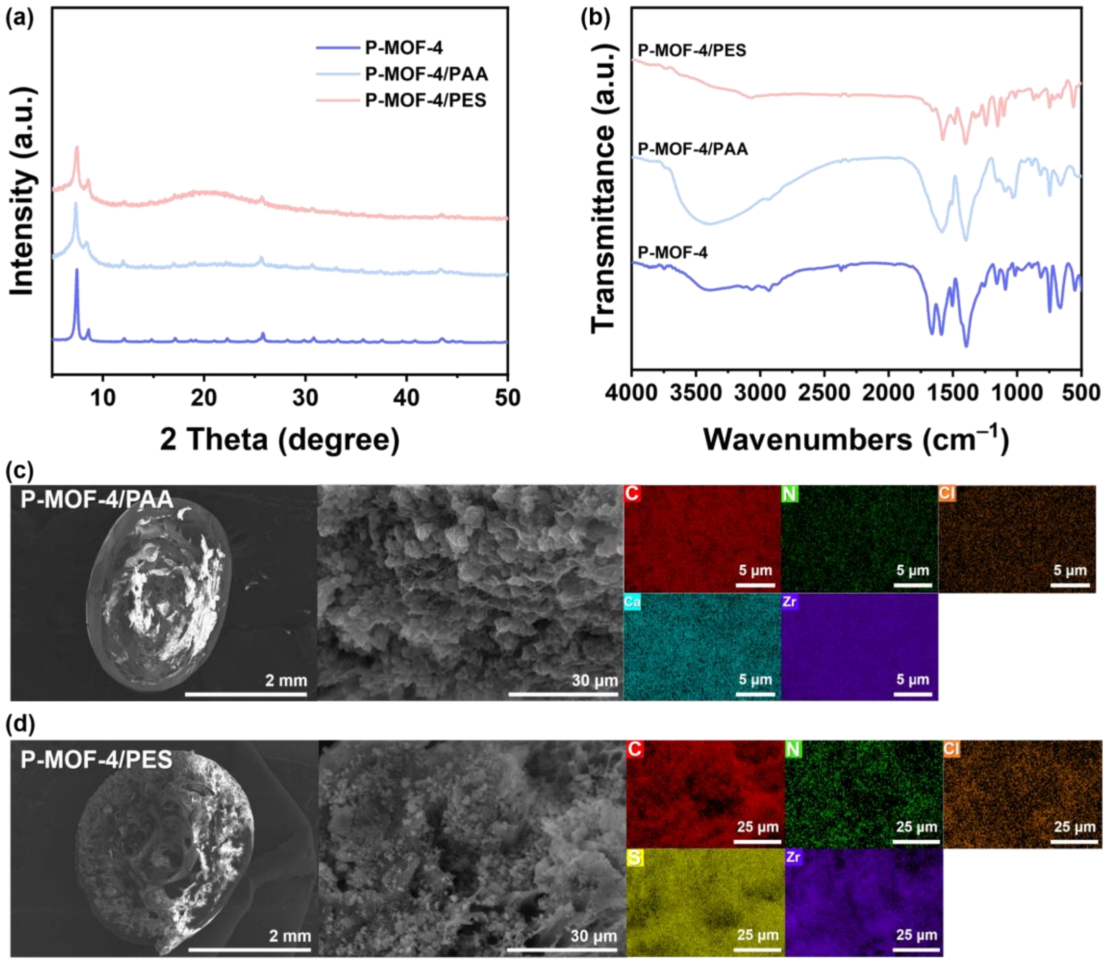

Figure S23. Characterizations of P-MOF-4 composite beads. (a) PXRD pattern and (b) FT-IR spectra of pristine P-MOF-4 powder, P-MOF-4/PAA bead, and P-MOF-4/PES bead. SEM image of the whole intersection and the closeup (including elemental mapping analysis) of (c) P-MOF-4/PAA bead and (d) P-MOF-4/PES bead.

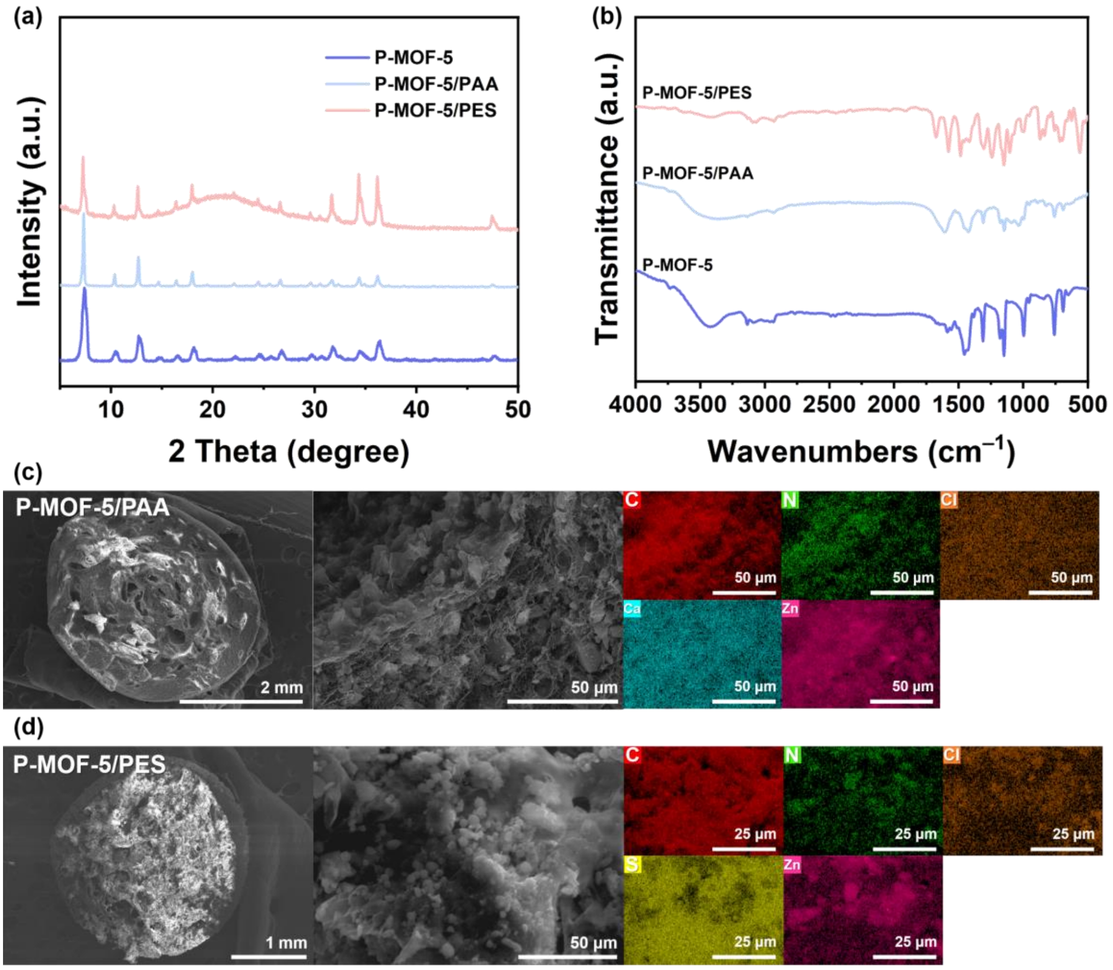

Figure S24. Characterizations of P-MOF-5 composite beads. (a) PXRD pattern and (b) FT-IR spectra of pristine P-MOF-5 powder, P-MOF-5/PAA bead, and P-MOF-5/PES bead. SEM image of the whole intersection and the closeup (including elemental mapping analysis) of (c) P-MOF-5/PAA bead and (d) P-MOF-5/PES bead.

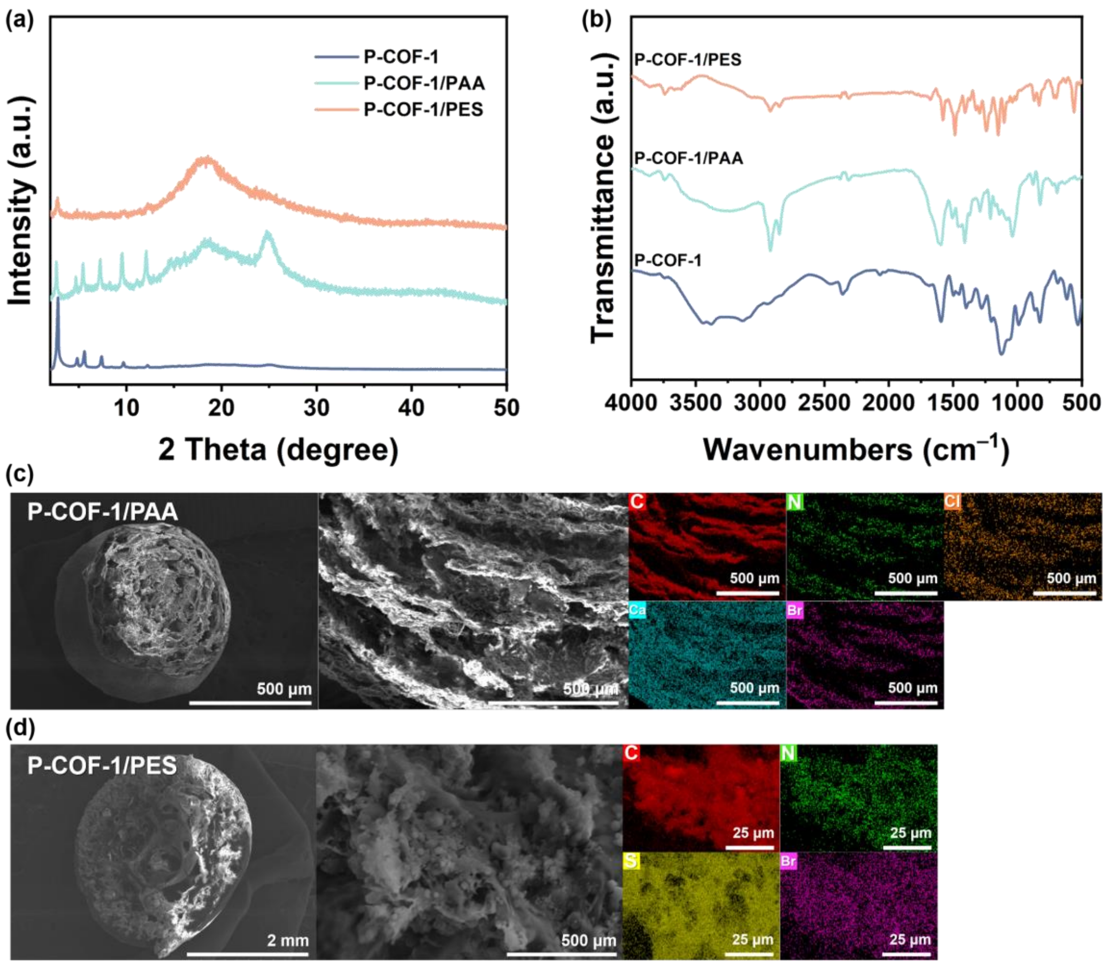

Figure S25. Characterizations of P-COF-1 composite beads. (a) PXRD pattern and (b) FT-IR spectra of pristine P-COF-1 powder, P-COF-1/PAA bead, and P-COF-1/PES bead. SEM image of the whole intersection and the closeup (including elemental mapping analysis) of (c) P-COF-1/PAA bead and (d) P-COF-1/PES bead.

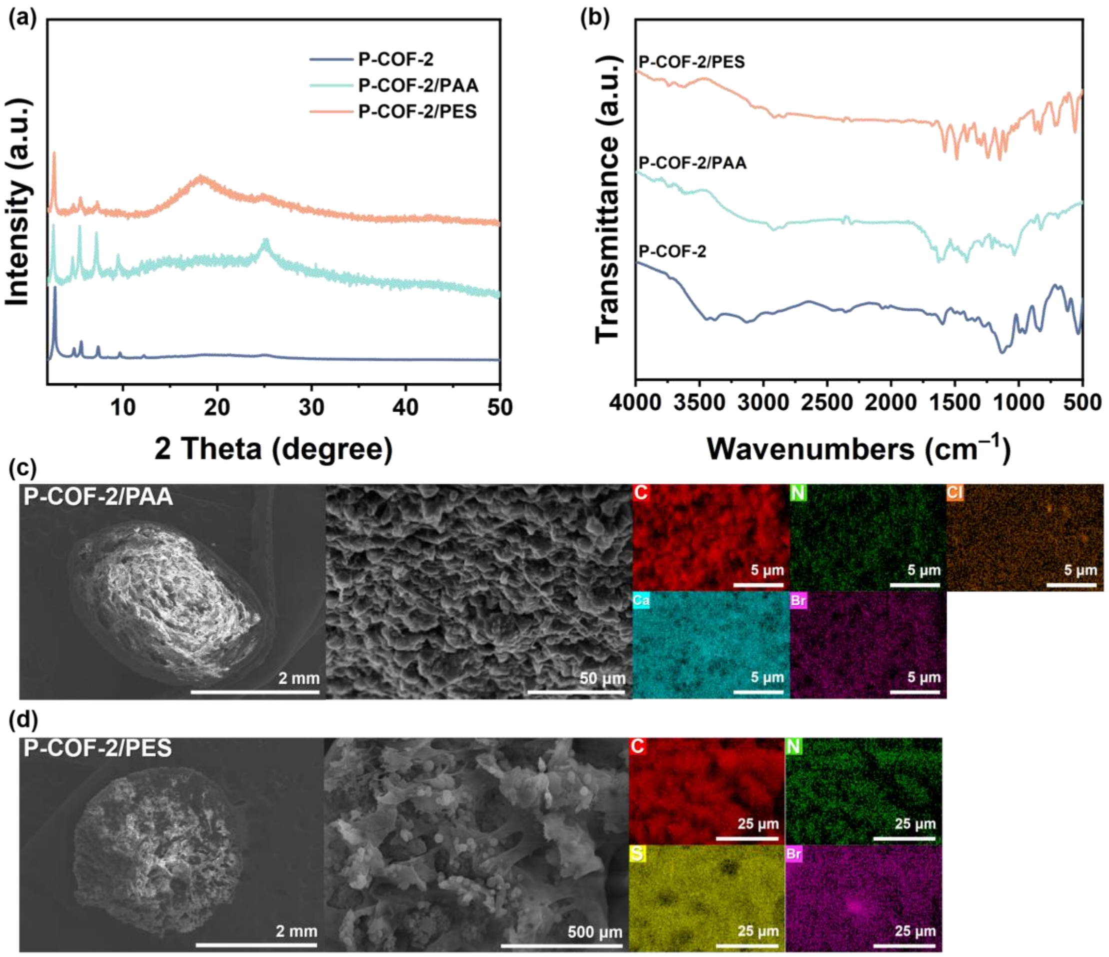

Figure S26. Characterizations of P-COF-2 composite beads. (a) PXRD pattern and (b) FT-IR spectra of pristine P-COF-2 powder, P-COF-2/PAA bead, and P-COF-2/PES bead. SEM image of the whole intersection and the closeup (including elemental mapping analysis) of (c) P-COF-2/PAA bead and (d) P-COF-2/PES bead.

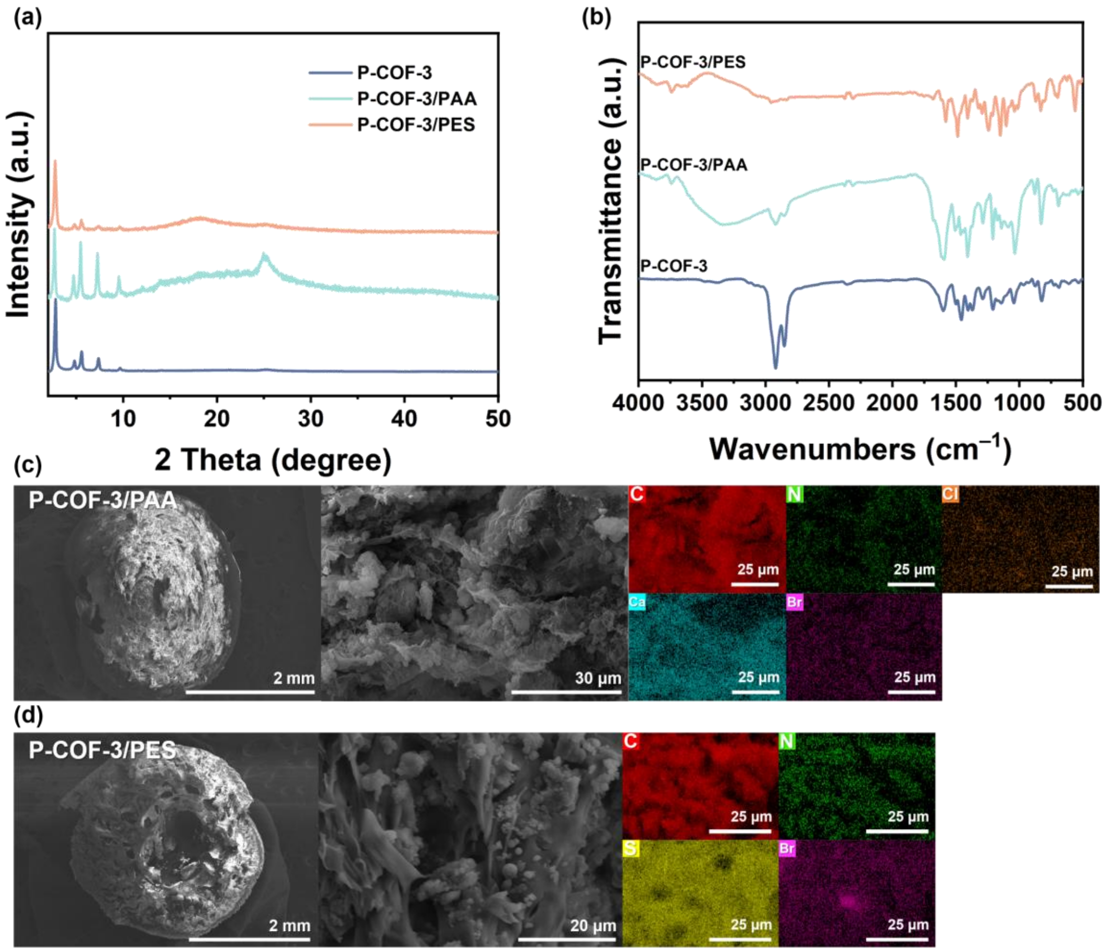

Figure S27. Characterizations of P-COF-3 composite beads. (a) PXRD pattern and (b) FT-IR spectra of pristine P-COF-3 powder, P-COF-3/PAA bead, and P-COF-3/PES bead. SEM image of the whole intersection and the closeup (including elemental mapping analysis) of (c) P-COF-3/PAA bead and (d) P-COF-3/PES bead.

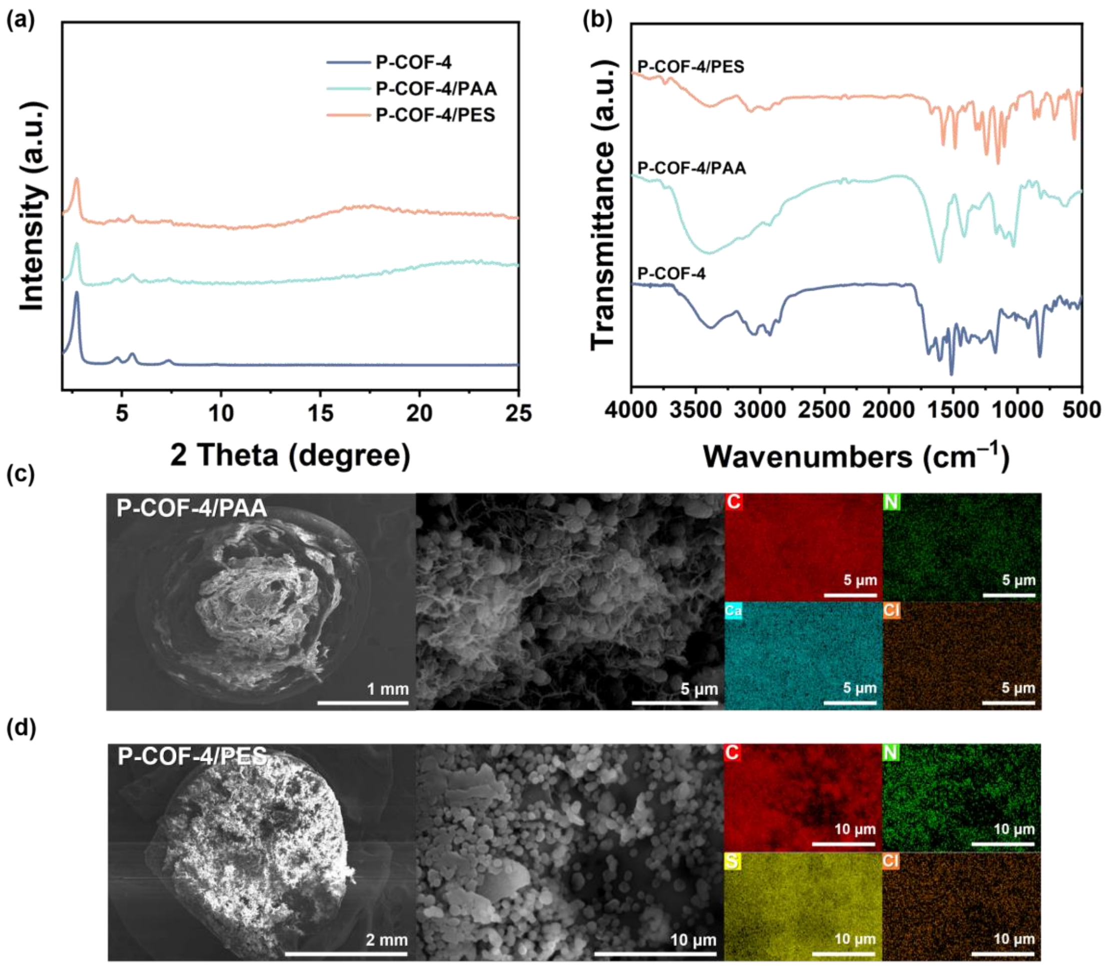

Figure S28. Characterizations of P-COF-4 composite beads. (a) PXRD pattern and (b) FT-IR spectra of pristine P-COF-4 powder, P-COF-4/PAA bead, and P-COF-4/PES bead. SEM image of the whole intersection and the closeup (including elemental mapping analysis) of (c) P-COF-4/PAA bead and (d) P-COF-4/PES bead.

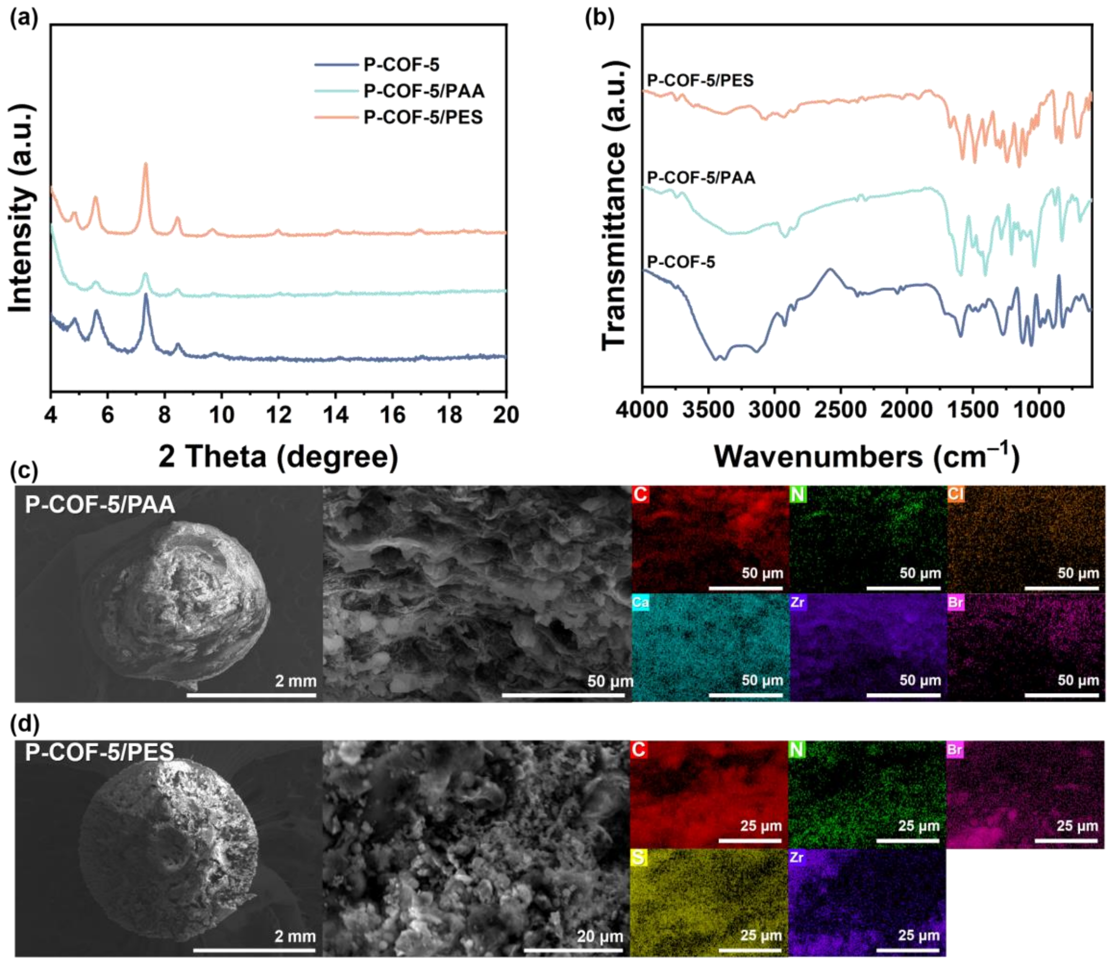

Figure S29. Characterizations of P-COF-5 composite beads. (a) PXRD pattern and (b) FT-IR spectra of pristine P-COF-5 powder, P-COF-5/PAA bead, and P-COF-5/PES bead. SEM image of the whole intersection and the closeup (including elemental mapping analysis) of (c) P-COF-5/PAA bead and (d) P-COF-5/PES bead.

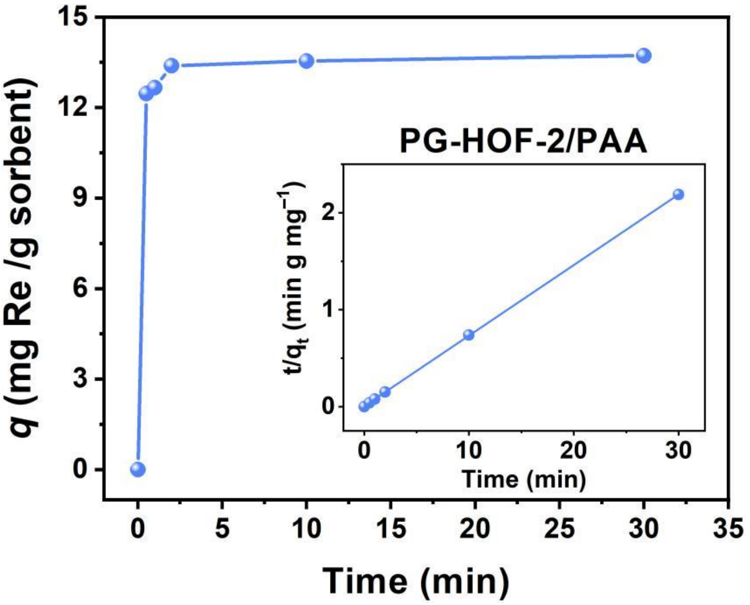

Figure S30. Sorption kinetics of PG-HOF-2/PAA for initially 25 ppm Re(VII). Inset: Pseudo-second-order model fitting.

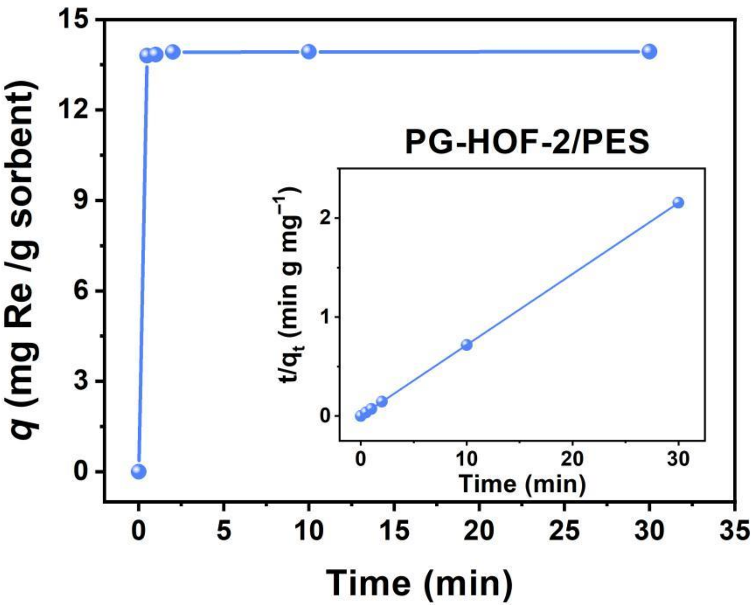

Figure S31. Sorption kinetics of PG-HOF-2/PES for initially 25 ppm Re(VII). Inset: Pseudo-second-order model fitting.

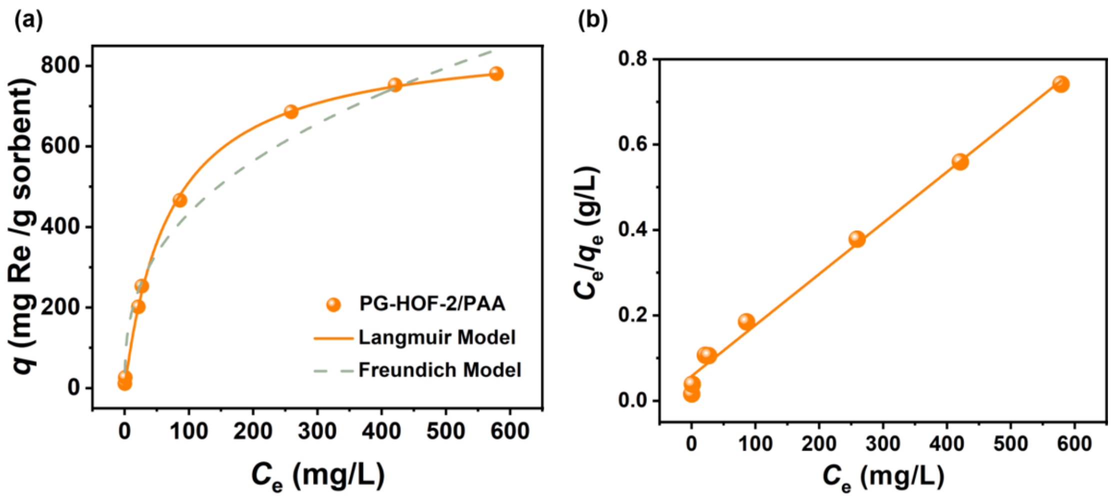

Figure S32. (a) Sorption isotherms of PG-HOF-2/PAA at pH = 7. (b) Linear regression by fitting the equilibrium data with the Langmuir sorption model.

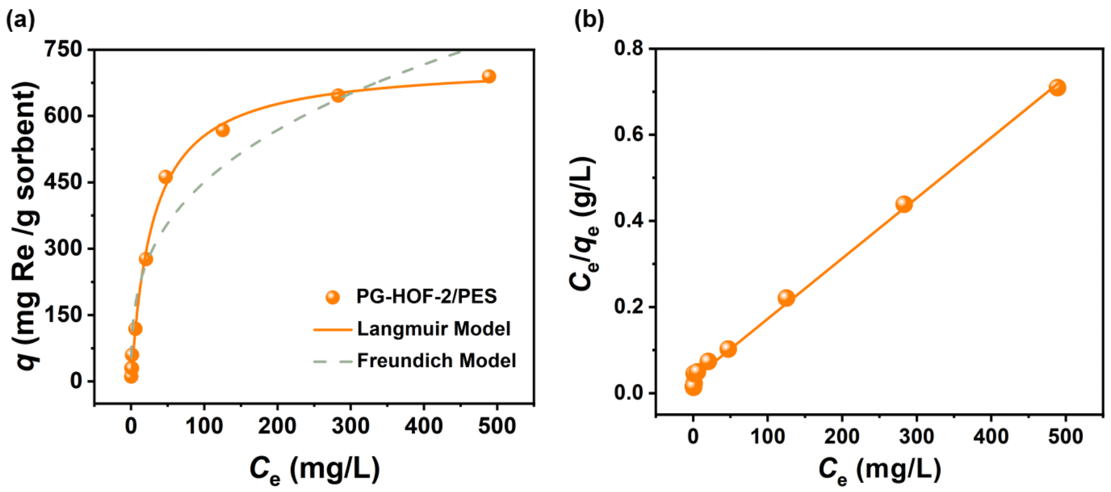

Figure S33. (a) Sorption isotherms of PG-HOF-2/PES at pH = 7. (b) Linear regression by fitting the equilibrium data with the Langmuir sorption model.

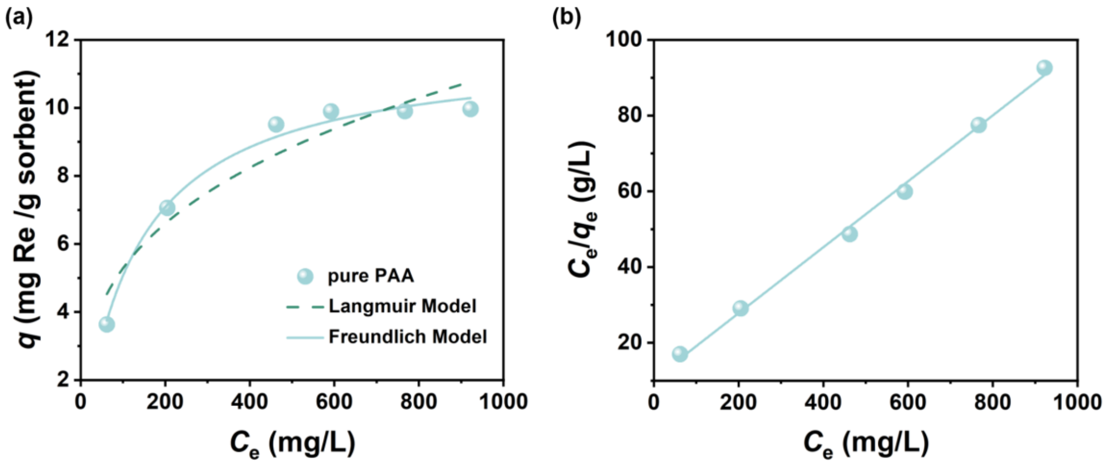

Figure S34. (a) Sorption isotherms of pure PAA at pH = 7. (b) Linear regression by fitting the equilibrium data with the Langmuir sorption model. The maximum uptake capacity for ReO_4^- was calculated to be 15.9 mg/g.

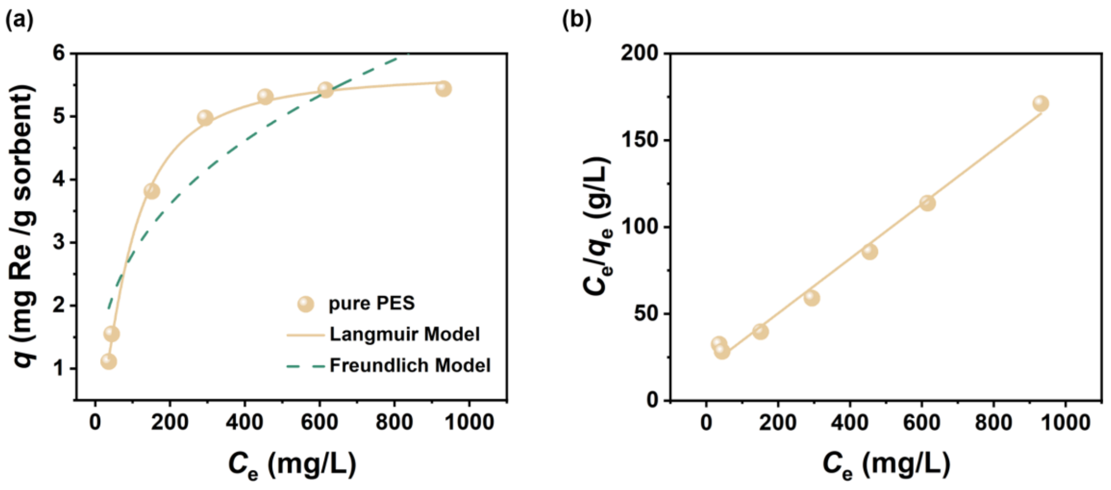

Figure S35. (a) Sorption isotherms of pure PES at pH = 7. (b) Linear regression by fitting the equilibrium data with the Langmuir sorption model. The maximum uptake capacity for ReO_4^- was calculated to be 7.7 mg/g.

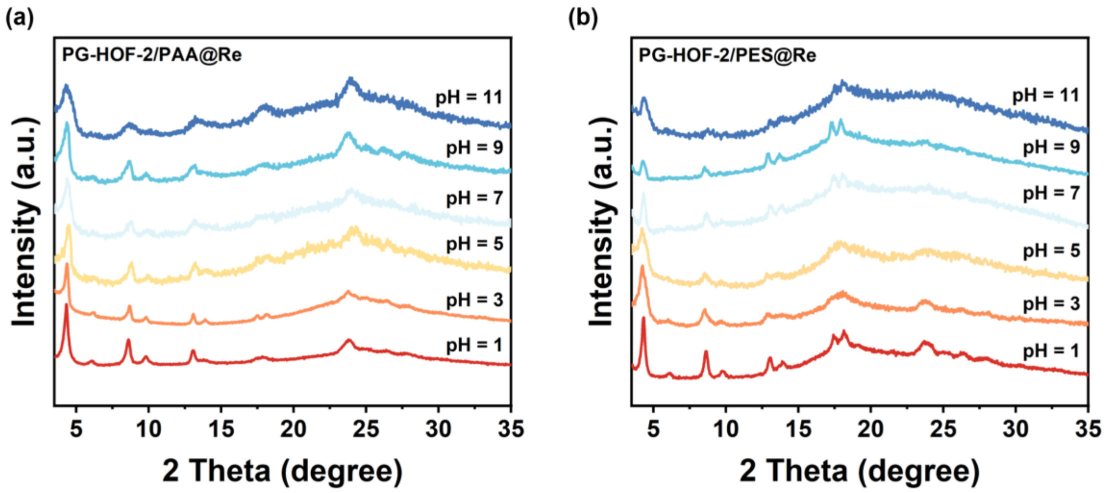

Figure S36. PXRD patterns of (a) PG-HOF-2/PAA and (b) PG-HOF-2/PES in ReO_4^- solutions at different pH conditions.

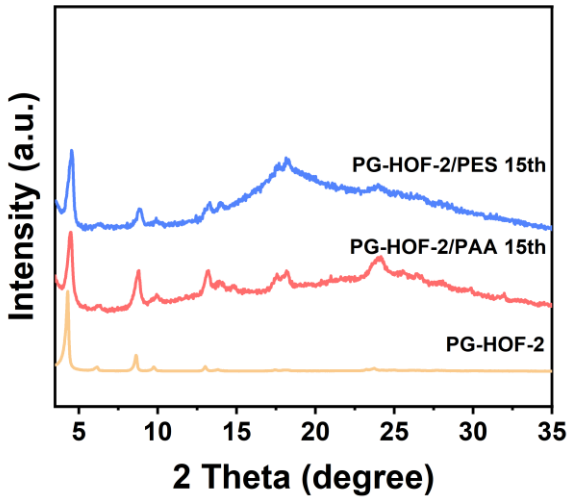

Figure S37. PXRD patterns of PG-HOF-2/PAA and PG-HOF-2/PES after fifteen cycles.

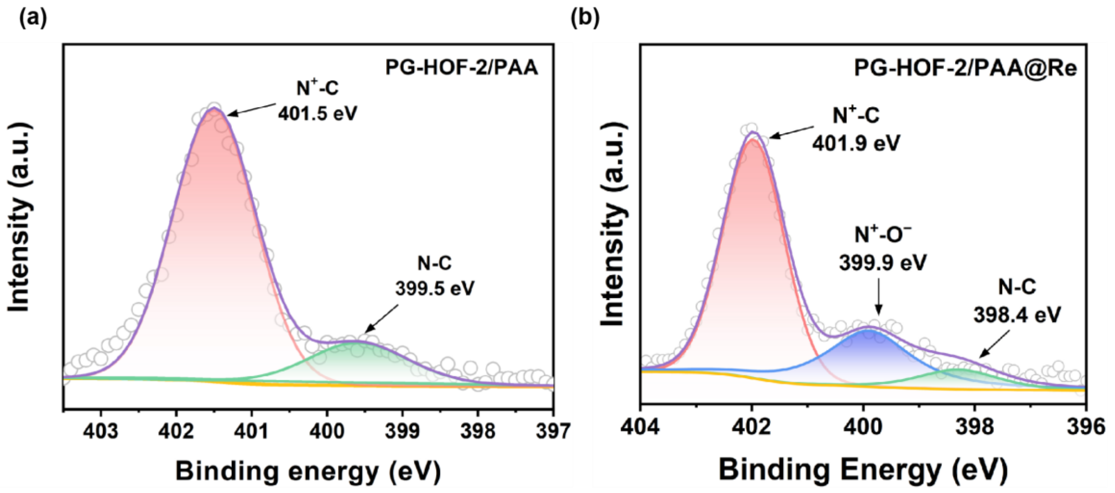

Figure S38. N 1*s* XPS spectra of (a) PG-HOF-2/PAA and (b) PG-HOF-2/PAA@Re.

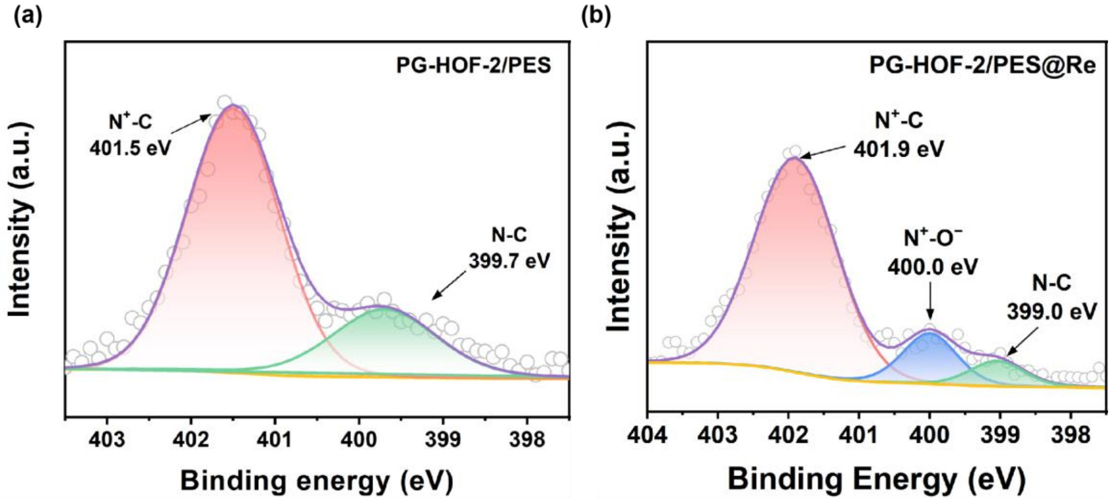

Figure S39. N 1*s* XPS spectra of (a) PG-HOF-2/PES and (b) PG-HOF-2/PES@Re.

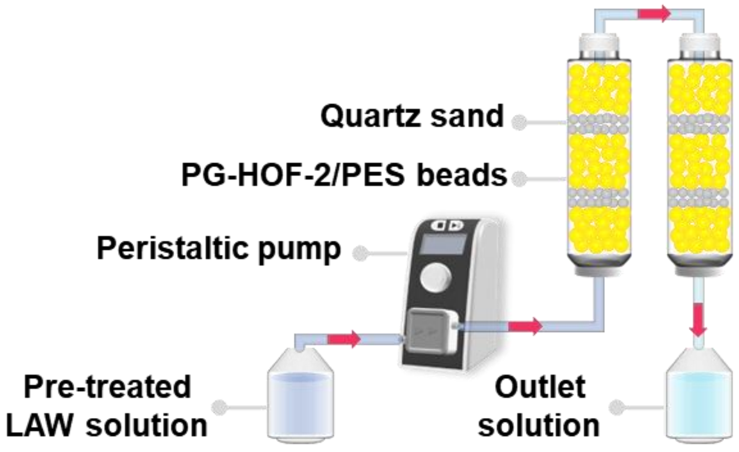

Figure S40. The diagram of the dynamic experiment system of PG-HOF-2/PES.

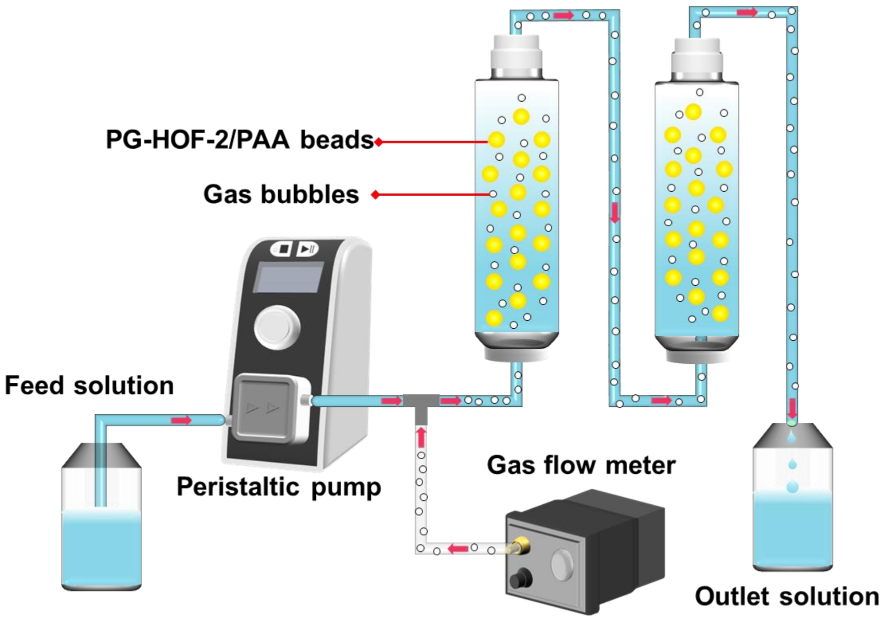

Figure S41. The process flow diagram of the gas-sparged adsorption system for PG-HOF-2/PAA beads.

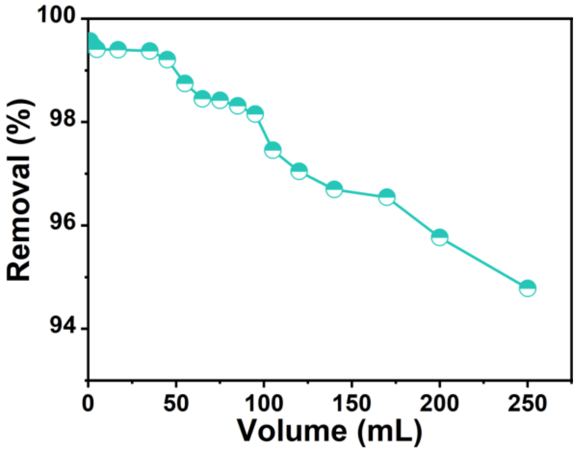

Figure S42. Removal efficiency of ReO_4^- by PG-HOF-2/PAA column.

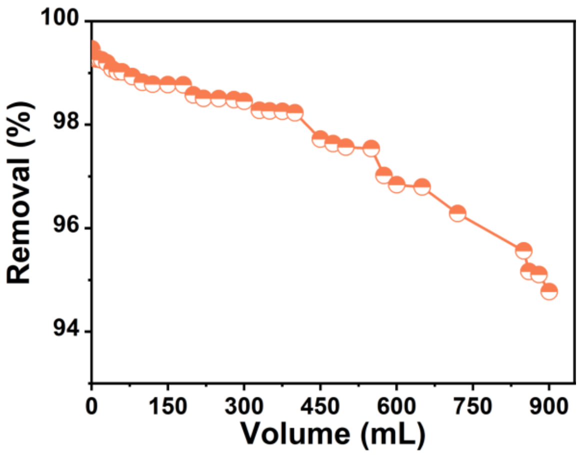

Figure S43. Removal efficiency of ReO_4^- by PG-HOF-2/PES column.

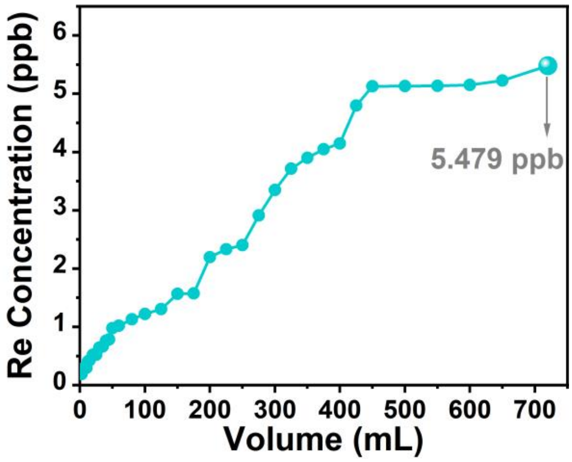

Figure S44. Dynamic sorption of ReO_4^- by PG-HOF-2/PAA for pre-treated LAW solution.

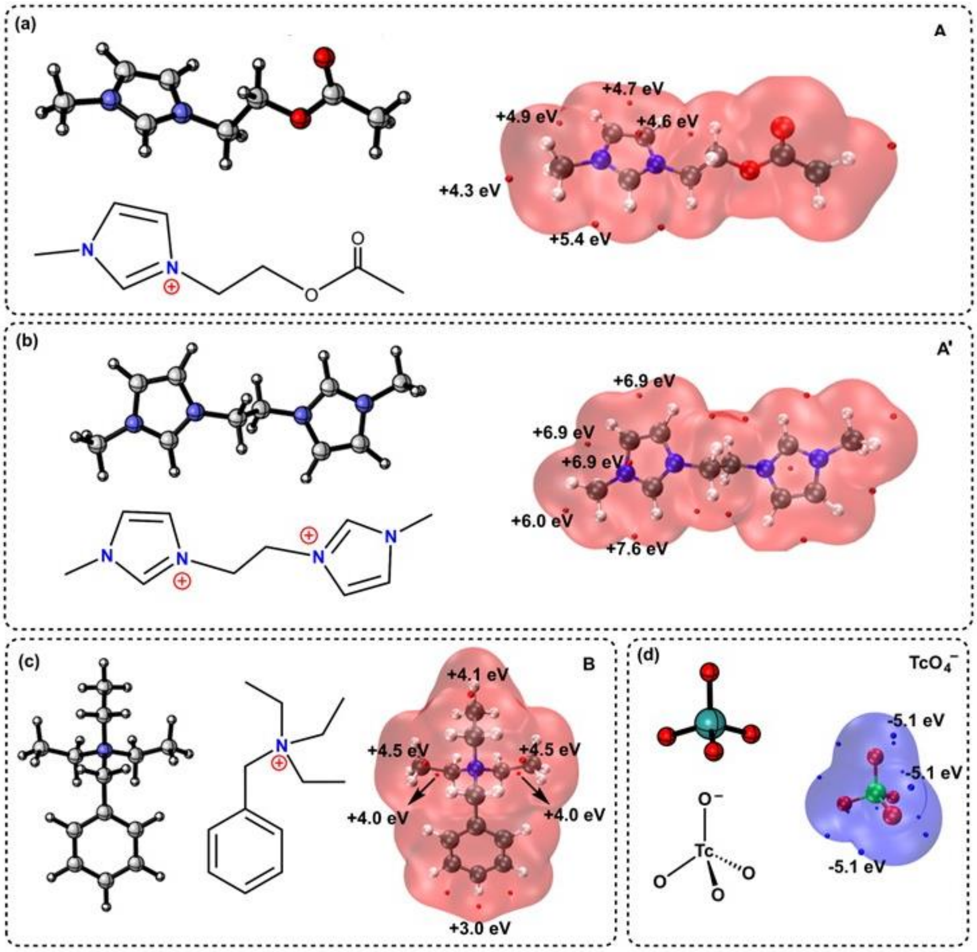

Figure S45. Optimized geometries and electrostatic potential (ESP)-mapped molecular vdW surfaces of (a) A, (b) A′, (c) B and (d) TcO_4^-. The red color shows positive negative electrostatic potentials, and the blue color shows negative electrostatic potentials.

Table S1. The BET surface areas and corresponding pore size distribution of PG-HOF-2, PG-HOF-2/PAA, and PG-HOF-2/PES.

Sample	BET Surface Area ($m^2\ g^{-1}$)	Pore Width (Å)	Pore Volume ($cm^3\ g^{-1}$)
PG-HOF-2	179.6	15.9	0.12
PG-HOF-2/PAA	85.3	14.8	0.04
PG-HOF-2/PES	24.6	14.8	0.02

Table S2. The removal percentages of ReO_4^- by PG-HOF-2, PG-HOF-2/PAA, and PG-HOF-2/PES at various intervals.

Time (min)	Removal of ReO_4^- (%)		
	PG-HOF-2	PG-HOF-2/PAA	PG-HOF-2/PES
0.5	97.93	89.33	98.92
1	98.01	90.76	99.20
2	98.29	95.94	99.80
10	98.57	97.07	99.88
30	99.34	98.37	99.89

Table S3. Comparison of sorption kinetics of PG-HOF-2/PAA and PG-HOF-2/PES with initially 25 ppm Re (VII).

Sorbent	q_e (mg g^{-1})	k_2 (g mg^{-1} min^{-1})	R^2
PG-HOF-2/PAA	13.74	1.19	0.9999
PG-HOF-2/PES	13.97	21.71	>0.9999

Table S4. Fitting results of ReO_4^- sorption by PG-HOF-2/PAA, PG-HOF-2/PES, PAA, and PES based on the Langmuir and Freundlich models.

Sorbent	Langmuir				Freundlich		
	q_m (mg Re g^{-1} sorbent)	q_m (mg ReO_4^- g^{-1} sorbent)	k_L (L mg^{-1})	R^2	k_F (L^n mol^{1-n} g^{-1})	n	R^2
PG-HOF-2/PAA	875.5	1176.7	0.014	0.999	76.8	2.7	0.973
PG-HOF-2/PES	720.1	967.9	0.034	0.996	96.4	3.0	0.942
PAA	11.8	15.9	0.008	0.989	1.2	3.1	0.916
PES	5.7	7.7	0.001	0.998	0.5	2.8	0.863

Table S5. Comparison of ReO_4^- sorption capacities across different sorbents.

Sorbent	Uptake capacity of ReO_4^- (mg g^{-1})	K_d (mL g^{-1})	Ref.
PG-HOF-1	1616	4.8×10^5	[5]
iCOP-1	1434	1.0×10^6	[18]
CPN-3	1282	7.0×10^7	[19]
**PG-HOF-2/PAA**	**1177**	$\mathbf{6.4 \times 10^4}$	**this work**
PG-HOF-2	1052	3.8×10^5	[5]
**PG-HOF-2/PES**	**968**	$\mathbf{1.4 \times 10^7}$	**this work**
polyILs@COF	946	2.7×10^4	[2]
NCE fibers	943	1.1×10^5	[20]
PMiMCl resin	909	1.3×10^5	[21]
PCE fibers	826	3.1×10^5	[22]
Purolite A530E	706	5.0×10^5	[20]
IRA-401	464	6.3×10^4	[20]
SCU-CPN-4	437	1.5×10^7	[23]
polyILs@MOF@COF	382	1.1×10^5	[4]
polyILs@MOF	362	3.3×10^6	[1]
CMS-*g*-$VBPPh_3NO_3$	272	2.3×10^6	[24]
Ag-TPEE	251	3.9×10^5	[25]
IM-WS	201	3.8×10^5	[20]
3DCOF-*g*-$VBPPh_3Cl$	181	1.0×10^8	[26]
GAC 830	28	1.1×10^5	[27]

Table S6. The removal efficiency of competing experiment by PG-HOF-2, PG-HOF-2/PAA, and PG-HOF-2/PES.

Anion	PG-HOF-2 (%)	PG-HOF-2/PAA (%)	PG-HOF-2/PES (%)
Blank	99.17	97.04	99.98
NO_3^-	98.02	94.41	99.76
NO_2^-	96.98	94.59	99.95
SO_4^{2-}	99.17	93.71	98.69
ClO_4^{2-}	97.39	93.04	98.82
PO_4^{3-}	99.33	93.03	99.63
CO_3^{2-}	99.75	97.23	98.87

Table S7. ReO_4^- Distribution Coefficients (K_d, mL g^{-1}) of PG-HOF-2, PG-HOF-2/PAA, and PG-HOF-2/PES after recycle (initial Re concentration: 451 ppb).

Samples	Residual concentration (ppb)	K_d (mL g^{-1})
PG-HOF-2 5th	0.036	3.16×10^5
PG-HOF-2 10th	0.041	2.79×10^5
PG-HOF-2 15th	0.039	2.98×10^5
PG-HOF-2/PAA5th	7.045	6.31×10^4
PG-HOF-2/PAA 10th	6.911	6.43×10^4
PG-HOF-2/PAA 15th	6.325	7.04×10^4
PG-HOF-2/PES 5th	1.426	1.25×10^7
PG-HOF-2/PES 10th	1.611	1.10×10^7
PG-HOF-2/PES 15th	1.510	1.16×10^7

Table S8. The XPS binding energy of PG-HOF-2, PG-HOF-2/PAA, PG-HOF-2/PES, PG-HOF-2/PAA@Re, and PG-HOF-2/PES@Re.

Samples	The binding energy (eV) of characteristic elements							
	Cl		Ca		S		Re	
	2*s*	2*p*	2*p*	3*p*	2*s*	2*p*	$4f_{5/2}$	$4f_{7/2}$
PG-HOF-2	267.9	197.2	-	-	-	-	-	-
PG-HOF-2/PAA	267.9	197.4	348.3	25.8	-	-	-	-
PG-HOF-2/PES	268.1	197.1	-	-	231.9	168.1	-	-
PG-HOF-2/PAA@Re	-	-	348.3	25.6	-	-	48.1	45.5
PG-HOF-2/PES@Re	-	-	-	-	232.8	167.6	48.3	45.9

Table S9. Composition of simulated Hanford LAW melter recycle stream.

Anion	Concentration (mol L^{-1})	Anion (molar ratio)
TcO_4^- (ReO_4^-)	1.94×10^{-4}	1
NO_3^-	6.07×10^{-2}	314
Cl^-	6.39×10^{-2}	330
NO_2^-	1.69×10^{-1}	873
SO_4^{2-}	6.64×10^{-6}	0.343
CO_3^{2-}	4.30×10^{-5}	0.222

Table S10. The removal performances of CPFs-X and CPFs-X/PES for pre-treated LAW solution (initial concentration of Re was 12.298 ppb).

Sorbent	Residual concentration (ppb)	
	powder	PES beads
P-MOF-1	0.371	0.248
P-MOF-2	1.808	1.15
P-MOF-3	1.207	0.812
P-MOF-4	2.144	1.378
P-MOF-5	1.025	0.718
P-COF-1	1.187	0.893
P-COF-2	1.124	0.787
P-COF-3	2.708	1.859
P-COF-4	1.945	1.125
P-COF-5	0.912	0.403
PG-HOF-1	0.171	0.093
PG-HOF-2	0.059	0.038
PG-HOF-3	0.082	0.047
PG-HOF-4	0.078	0.056
PG-HOF-5	0.111	0.095
PG-HOF-6	0.091	0.059
PG-HOF-7	0.339	0.180

References

[1] C.-P. Li, H.-R. Li, J.-Y. Ai, J. Chen, M. Du, *ACS Cent. Sci*. **2020**, *6*, 2354–2361.

[2] Z.-F. Liu, K. Liu, X.-J. Zheng, Y.-H. Wang, X.-X. Sun, P.-C. Xue, C.-P. Li, M. Du, *Chem. Mater*. **2022**, *34*, 5452–5460.

[3] L. Zhu, H.-R. Li, Z.-F. Liu, Z. Di, W. Xu, L. Zhang, C.-P. Li, *Chem. Eur. J*. **2023**, *29*, e202302168.

[4] Z. Di, Z.-F. Liu, H.-R. Li, Z. Liu, C.-P. Li, *Inorg. Chem. Front.* **2023**, *10*, 952–958.

[5] H.-R. Li., S. W., X. Jing, Z. Di, K. Liu, L. Wang, C.-P. Li, Z. Liu, M. Du, *Sci. China Chem*. **2024**, *67*, 2958–2967.

[6] F. Hu, C. Liu, M. Wu, J. Pang, F. Jiang, D. Yuan, M. Hong, *Angew. Chem. Int. Ed*. **2017**, *56*, 2101–2104.

[7] Q. Yin, J. Lü, H.-F. Li, T.-F. Liu, R. Cao, *Cryst. Growth Des*. **2019**, *19*, 4157–4161.

[8] G. Liu, M. Hou, J. Song, T. Jiang, H. Fan, Z. Zhang, B. Han, *Green Chem*. **2010**, *12*, 65–69.

[9] W.-L. Dai, B. Jin, S.-L. Luo, S.-F. Yin, X.-B. Luo, C.-T. Au, *J. CO_2 Util*. **2013**, *3–4*, 7–13.

[10] E. S. Domnina, L. V. Baikalova, D. D. Taryashinova, N. N. Chipanina, V. K. Voronov, G. G. Skvortsova, *Bull. Acad. Sci. USSR, Div. Chem. Sci*. **1979**, *28*, 1931–1936.

[11] Y. Zhao, D. G. Truhlar, *Theor. Chem. Acc*. **2008**, *120*, 215–241.

[12] S. Grimme, J. Antony, S. Ehrlich, H. Krieg, *J. Chem. Phys*. **2010**, *132*, 154104.

[13] Y. Matsuo, E. Nakamura, *Organometallics* **2003**, *22*, 2554-2563.

[14] Gaussian 16, Revision B.01, M. J. Frisch, G. W. Trucks, H. B. Schlegel, G. E. Scuseria, M. A. Robb, J. R. Cheeseman, G. Scalmani, V. Barone, G. A. Petersson, H. Nakatsuji, X. Li, M. Caricato, A. V. Marenich, J. Bloino, B. G. Janesko, R. Gomperts, B. Mennucci, H. P. Hratchian, J. V. Ortiz, A. F. Izmaylov, J. L. Sonnenberg, Williams, F. Ding, F. Lipparini, F. Egidi, J. Goings, B. Peng, A. Petrone, T. Henderson, D. Ranasinghe, V. G. Zakrzewski, J. Gao, N. Rega, G. Zheng, W. Liang, M. Hada, M. Ehara, K. Toyota, R. Fukuda, J. Hasegawa, M. Ishida, T. Nakajima, Y. Honda, O. Kitao, H. Nakai, T. Vreven, K. Throssell, J. A. Montgomery Jr., J. E. Peralta, F. Ogliaro, M. J. Bearpark, J. J. Heyd, E. N. Brothers, K. N. Kudin, V. N. Staroverov, T. A. Keith, R. Kobayashi, J. Normand, K. Raghavachari, A. P. Rendell, J. C. Burant, S. S. Iyengar, J. Tomasi, M. Cossi, J. M. Millam, M. Klene, C. Adamo, R. Cammi, J. W. Ochterski, R. L. Martin, K. Morokuma, O. Farkas, J. B. Foresman, D. J. Fox, Gaussian, Inc. Wallingford, CT, **2016**.

[15] T. Lu, F. Chen, *J. Comput. Chem*. **2012**, *33*, 580–592.

[16] F. Weigend, R. Ahlrichs, *Phys. Chem. Chem. Phys*. **2005**, *7*, 3297–3305.
[17] A. V. Marenich, C. J. Cramer, D. G. Truhlar, *J. Phys. Chem. B* **2009**, *113*, 6378–6396.
[18] X. Yang, W. Wu, Y. Xie, M. Hao, X. Liu, Z. Chen, H. Yang, G. I. N. Waterhouse, S. Ma, X. Wang, *Environ. Sci. Technol*. **2023**, *57*, 10870–10881.
[19] B. Wang, J. Li, H. Huang, B. Liang, Y. Zhang, L. Chen, K. Tan, Z. Chai, S. Wang, J. T. Wright, R. W. Meulenberg, S. Ma, *ACS Cent. Sci*. **2024**, *10*, 426–438.
[20] D. Chen, Z. Liu, S. Li, X. Jing, Y. Tian, W. Hu, F. Cui, R. Zhao, G. Zhu, *Chem. Eng. J*. **2023**, *452*, 139148.
[21] T. Hu, W. Hao, H. Zou, W. Xue, D. Mei, Y. Song, W. Yan, *Chem. Eng. J.* **2023**, *465*, 142951.
[22] R. Zhao, D. Chen, N. Gao, L. Yuan, W. Hu, F. Cui, Y. Tian, W. Shi, S. Ma, G. Zhu, *Adv. Funct. Mater*. **2022**, *32*, 2200618.
[23] J. Li, B. Li, N. Shen, L. Chen, Q. Guo, L. Chen, L. He, X. Dai, Z. Chai, S. Wang, *ACS Cent. Sci*. **2021**, *7*, 1441–1450.
[24] Y. Wang, D. Han, S. Zhong, X. Li, H. Su, T. Chu, J. Peng, L. Zhao, J. Li, M. Zhai, *J. Hazard. Mater.* **2021**, *401*, 123354.
[25] K. Kang, S. Liu, M. Zhang, L. Li, C. Liu, L. Lei, X. Dai, C. Xu, C. Xiao, *Adv. Funct. Mater*. **2022**, *32*, 2208148.
[26] H.-J. Da, C.-X. Yang, X.-P. Yan, *Environ. Sci. Technol*. **2019**, *53*, 5212–5220.
[27] D. Li, J. C. Seaman, D. I. Kaplan, S. M. Heald, C. Sun, *Chem. Eng. J.* **2019**, *360*, 1–9.
